# Unveiling Temperature-Induced Structural Phase Transformations and CO_2_ Binding Sites in CALF-20

**DOI:** 10.1021/acs.inorgchem.4c02952

**Published:** 2024-09-27

**Authors:** Joanna Drwęska, Filip Formalik, Kornel Roztocki, Randall Q. Snurr, Leonard J. Barbour, Agnieszka M. Janiak

**Affiliations:** † Faculty of Chemistry, Adam Mickiewicz University, Uniwersytetu Poznańskiego 8, 61-614 Poznań, Poland; ‡ Department of Micro, Nano, and Bioprocess Engineering, Faculty of Chemistry, Wroclaw University of Science and Technology, Wybrzeże Wyspiańskiego 27, 50-370 Wrocław, Poland; § Department of Chemical and Biological Engineering, Northwestern University, Evanston, Illinois 60208, United States; ∥ Department of Chemistry and Polymer Science, Stellenbosch University, Private Bag X1, Matieland 7602, South Africa

## Abstract

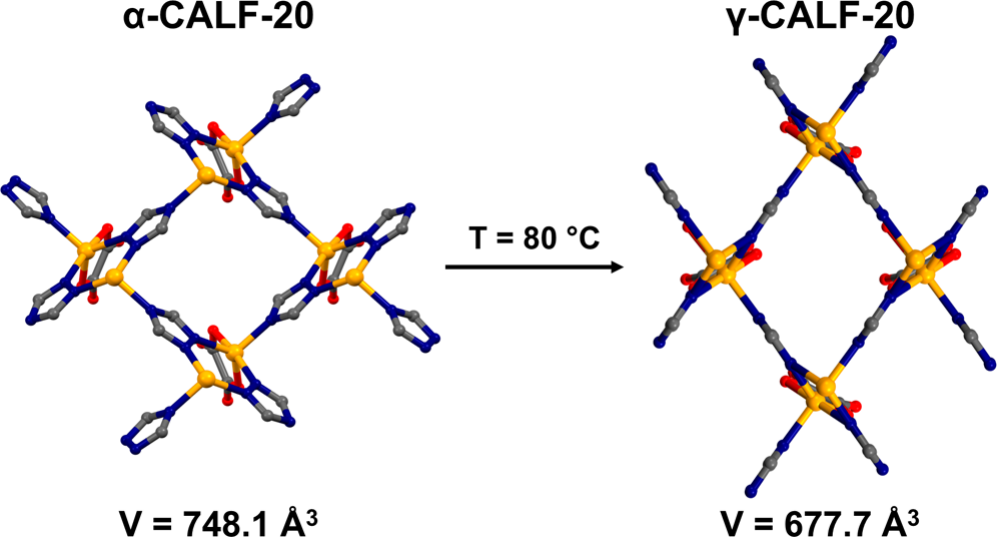

The increase in atmospheric carbon dioxide concentration linked to climate change has created a need for new sorbents capable of separating CO_2_ from exhaust gases. Recently, an easily produced metal–organic framework, CALF-20, was shown to withstand over 450,000 adsorption/desorption cycles in steam and wet acid gases. Further development and industrial application of such materials require an understanding of the observed processes. Herein, we demonstrate that conditioning as-synthesized CALF-20 single crystal transforms it into a different phase, γ-CALF-20. The transformation resulted in significant structural changes, including the binding of water molecules to Zn(II), accompanied by a reduction of 9% in the unit cell volume. Our experimental findings were supported by the energy-volume dependence of CALF-20 in the presence and absence of water molecules calculated from density functional theory. We have also monitored the sorption process of the dominant greenhouse gas, CO_2_, on the initial phase of CALF-20 at atomic resolution using in situ single-crystal X-ray diffraction under specific pressure. The new understanding of CALF-20 chemistry from these studies should facilitate development of novel sorbents for gas adsorption technologies.

## Abstract 2

We have found a single-crystal-to-single-crystal phase transition in CALF-20 metal−organic framework. Theoretical analysis of the various CALF-20 phases has provided valuable insights into the observed processes. By conducting an in situ single-crystal X-ray diffraction experiment under controlled CO_2_ gas pressure, we were able to experimentally determine the carbon dioxide positions within the framework at the atomic level, allowing us to gain better understanding on the nature of CALF-20-CO_2_ interactions.

## Introduction

In 2022, the National Oceanic and Atmospheric Administration recorded an average atmospheric carbon dioxide level of 417 ppm, a 50% increase from preindustrial levels of 280 ppm.^1^ This rapid increase in greenhouse gas concentration is causing serious consequences for both humans and the environment.^2,3^ The Intergovernmental Panel on Climate Change has identified several impacts of global warming in its Sixth Assessment Report, including coral reef extinction, melting of polar ice caps, glacier shrinkage, persistent drought, and deadly heat waves.^4^ To address this issue, 196 countries signed the Paris Agreement, which provides a framework for international climate policy.^5^ The agreement calls for signatory countries to limit the global average temperature increase to less than 2 °C above preindustrial levels, and preferably 1.5 °C. However, human activity accounted for the emission of a record 40.5 billion tonnes of carbon dioxide in 2022.^6^ Given that fossil fuels will continue to be a significant source of CO_2_ emissions in the foreseeable future, achieving zero net carbon dioxide emissions^7,8^ will require the development of versatile carbon dioxide capture technologies.^9,10^

Recently, metal–organic frameworks (MOFs), coordination polymers containing potential voids,^11^ have emerged as a class of sorbents that can remove dilute CO_2_ from flue gas emitted by stationary sources.^12,13^ However, many MOFs do not meet the necessary requirements of stability, scalability, processability, and regenerability that would provide a sufficient lifetime at a reasonable production cost. For example, amine-functionalized Mg-MOF-74 shows promising CO_2_ cooperative adsorption behavior,^14^ enabling efficient CO_2_ capture under desired conditions, and the material has demonstrated excellent stability during temperature swing adsorption cycles, maintaining its total capacity up to 1000 cycles, indicating its strong regenerability.^15^ However, the preparation of this material requires a two-step process that includes synthesis and functionalization.

Among the thousands of known MOFs, CALF-20 (Calgary framework 20) has emerged as a promising sorbent for CO_2_ capture, with its durability proven over 450,000 cycles in steam and wet acid gases.^16^ Furthermore, CALF-20 can be synthesized in a one-step reaction in 50% ethanol as well as in a methanol aqueous solution at room temperature.^17^ This MOF easily forms composite materials and is multiton scalable. Interesting properties of CALF-20 have sparked a surge of interest in the framework, leading to both experimental and theoretical studies of its sorption capacities.^18−20^ It was determined that carbon dioxide and water compete for the same sites within the framework,^18^ which undergoes structural changes triggered by both of these substances.^20^ Flexibility of this MOF was also proven by comparing powder X-ray diffraction data of CALF-20 subjected to various external stimuli such as water soaking, water vapor, and nitrogen.^20^ Attempts at enhancing its sorption capabilities have also been studied.^21,22^ Another focus of research on this framework was its susceptibility to different external factors.^23−25^ A detailed understanding of the chemistry of CALF-20 is essential for its potential applications,^26−28^ as well as for further development of even more effective materials for carbon dioxide removal technologies. In this work, we present evidence that CALF-20 undergoes temperature-induced single-crystal-to-single-crystal transformations. As a result of these transformations, we discovered two new CALF-20 phases. Notably, aside from the original CALF-20, the new phases reported in this paper are the only ones obtained to date in single crystal form. Our experimental findings were supplemented by computational investigations that support the experimental results. Finally, by utilizing a state-of-the-art in situ single-crystal X-ray diffraction (SC-XRD) technique under carbon dioxide atmosphere, we located the CO_2_ binding site in the initial phase with atomic precision.

## Experimental Methods

### General Remarks

All reagents (except for zinc oxalate) and solvents were used as received from commercial sources without further purification. Unless otherwise stated, all manipulations were done under ambient conditions.

### Synthesis of Zinc Oxalate Dihydrate

Zn(NO_3_)_2_·6H_2_O (9.70 g, 32.6 mmol) and H_2_C_2_O_4_ (2.93 g, 32.6 mmol) were separately dissolved in distilled water to form homogeneous solutions. The solutions were then mixed thoroughly under vigorous stirring until a white ZnC_2_O_4_·2H_2_O precipitate was formed. The precipitate was washed several times with distilled water and dried in an air oven at 150 °C for 3 h.

### Synthesis of α-CALF-20 Single Crystals

The MOF was obtained according to the previously published procedure.^16^ Zn(NO_3_)_2_·6H_2_O (180.0 mg, 0.605 mmol) and 1,2,4-triazole (50.0 mg, 0.725 mmol) were dissolved in 50% ethanol (6.00 mL). 2,5-Dihydroxy-1,4-benzoquinone (50.0 mg, 0.357 mmol) was also dissolved in the same volume of 50% ethanol. The solutions were combined and stirred continuously for 5 min. The mixture was filtered on a Büchner funnel and 6 mL of the filtrate was transferred to a 20 mL Teflon lined autoclave. The autoclave was placed in an oven at 180 °C for 48 h and then the reaction system was slowly cooled to room temperature over 24 h. Colorless block-shaped crystals were collected. The molecular formula derived from the X-ray structure analysis is Zn_2_C_6_H_4_N_6_O_4_ = [Zn_2_(ox)(trz)_2_]_*n*_.

*Caution!* Extreme care should be taken in the handling of the autoclave due to the generation of high pressure inside.

### Activation of α-CALF-20 Single Crystals

Single crystal of α-CALF-20 was glued to a glass fiber and placed in the glass capillary. Subsequently, the sample was subjected to a vacuum at 80 °C for 3 h. The activation process was monitored by SC-XRD analysis, which revealed a notable decrease in electron density in the pores, shown by a reduction in electron count from 20 to 10. This reduction is directly linked to the removal of solvent molecules from the asymmetric unit (ASU) cell, confirming the formation of the activated phase called α-CALF-20-act.

*Caution!* Extreme care should be taken in the handling of the apparatus when vacuum is applied in order to avoid implosion.

### Preparation of γ-CALF-20 Single Crystals

α-CALF-20 single crystals were deposited on a Petri dish, which was placed uncovered in an oven at 80 °C for 10 days. The molecular formula derived from the X-ray structure analysis is Zn_2_C_6_H_4_N_6_O_5_ = [Zn_2_(ox)(trz)_2_(H_2_O)]_*n*_.

### Preparation of τ-CALF-20 Single Crystals

The crystals were obtained after heating α-CALF-20 crystals under the same conditions as γ-CALF-20, but for 7 days. The molecular formula derived from the X-ray structure analysis is Zn_2_C_6_H_4_N_6_O_5_ = [Zn_2_(ox)(trz)_2_(H_2_O)]_*n*_.

### Single Crystal X-ray Diffraction

Crystals were mounted on nylon CryoLoops with Paraton-N. Reflection intensities for α-CALF-20 were collected on a Bruker D8 QUEST diffractometer equipped with a Cu IμS DIAMOND II microfocus source (λ = 1.54178 Å) and a PHOTON III 7 detector. Data collection, data reduction, and multiscan absorption collection were performed with the APEX5 software.^29^ Reflection intensities for τ-CALF-20 and γ-CALF-20 were collected on a Rigaku SuperNova diffractometer equipped with a Cu microfocus source and a 135 mm Atlas CCD detector. If necessary, the sample temperature was controlled with an Oxford Instruments Cryojet Controller. Data collection, data reduction, and multiscan absorption collection were performed with the CrysAlisPro software.^30^ All crystal structures were solved by direct methods using SHELXT^31^ and refined by the full-matrix least-squares techniques with SHELXL^32^ within the graphical user interface X-Seed.^33^ Non-hydrogen atoms were refined anisotropically. Hydrogen atoms were located from difference Fourier maps and refined isotropically, with the exception of those in γ-CALF-20. Hydrogen atoms in γ-CALF-20 were placed at calculated positions and refined using riding models, with their isotropic displacement parameters assigned values 20% higher than the isotropic equivalent for the atoms to which they are attached. In τ-CALF-20 and γ-CALF-20, the water molecule coordinated to the Zn^2+^ cation was refined with fixed occupancy of 0.5. Due to the fractional site occupancy for coordinated water, its hydrogen atoms have not been included.

When the assignment of electron density to solvent molecules was highly unreliable, their contributions were subtracted from the diffraction data using SQUEEZE.^34,35^ SQUEEZE analysis determined the electron count to be 20 e̅/ASU in the accessible void volume of 80 Å^3^ in α-CALF-20, 8 e̅/ASU in the accessible void volume of 42 Å^3^ in τ-CALF-20, and 4 e̅/ASU in an accessible void volume of 37 Å^3^ in γ-CALF-20.

### Single Crystal X-ray Diffraction under CO_2_ Pressure

X-ray experiments performed on a crystal under controlled atmospheres were carried out in an environmental gas cell developed in-house by prof. Barbour.^36^ The gas cell consisted of a glass capillary attached to a stainless-steel tube with an inlet/outlet valve through which the gas was introduced or the vacuum was applied. The system was sealed from the atmosphere. The crystal was glued to a glass fiber and placed in the glass capillary. The X-ray measurements were performed for both the crystal under reduced (vacuum) and gas (CO_2_) pressure. Intensity data were collected on a Bruker D8 QUEST diffractometer equipped with a Cu IμS DIAMOND II microfocus source (λ = 1.54178 Å) and a PHOTON III 7 detector. Data collection, data reduction, and multiscan absorption collection were performed with the APEX5 software.^29^ The crystal structures were solved by direct methods using the SHELXT^31^ program and refined by full-matrix least-squares techniques with SHELXL^32^ through the graphical interface X-Seed.^33^ Non-hydrogen atoms were refined anisotropically except for those constituting the disordered model of the carbon dioxide, which were refined isotropically. The hydrogen atoms were placed at calculated positions and refined using riding models, and their isotropic displacement parameters were assigned values 20% higher than the isotropic equivalent for the atoms to which they are attached.

*Caution!* Extreme care should be taken in the handling of the apparatus when vacuum is applied in order to avoid implosion.

In α-CALF-20-CO_2_, the CO_2_ molecule is disordered over two symmetry related positions, with occupancy of 0.25 for each component of the disorder. In all disorder models, the restraints were applied: 1,2-distances related to bond lengths and 1,3-distances related to bond angles to keep them within a certain range during the refinement process. The site occupancy for CO_2_ molecules was initially determined to be 0.5, based on the indication of their atomic thermal factors and later confirmed through electron density summation during SQUEEZE analysis. The activated form contained 10 electrons per ASU, which increased to 20 e̅/ASU under CO_2_ pressure. This suggests the squeezing of approximately 0.45 molecule of CO_2_ per ASU (10/22∼0.45).

Molecular graphics images were produced either within X-Seed^33^ using Pov-Ray^37^ or Mercury.^38^

Fourier transform infrared (FT-IR) spectra were detected on a Jasco FT/IR-4600 FT-IR Spectrometer in the range of 4000–400 cm^–1^ using a single-reflection ATR attachment.

Powder X-ray diffraction (P-XRD) patterns were measured at room temperature on a Bruker D8 Advance powder diffractometer equipped with a Johansson monochromator (λ = 1.54178 Å) and a f40 silicon strip detector LynxEye or on a Rigaku SuperNova diffractometer equipped with Cu microfocus source and 135 mm Atlas CCD detector. The patterns were visualized using Origin(Pro).^39^

Simultaneous thermal analyses (STA) were performed on a PerkinElmer STA 6000 Simultaneous Thermal Analyzer at a heating rate of 10 °C min^–1^ in a temperature range of 23–994 °C (approximately sample weight of 10 mg). The measurements were carried out at atmospheric pressure.

### Theoretical Calculations

To study structural changes in CALF-20 we employed the density functional theory (DFT) method as implemented in VASP.^40−42^ The Perdew–Burke–Ernzerhof (PBE) correlation-exchange functional^43^ was applied together with D3(BJ) dispersion correction.^44,45^ The plane-wave cutoff was set to 900 eV to ensure that volume changes do not affect the calculated energies, and a *k*-point grid was set to 3 × 3 × 3. The electronic steps convergence was set to 10^–6^ eV. Three types of geometry optimization were considered in this work: ionic positions (ISIF = 2), ionic positions and cell shape with fixed volume (ISIF = 4), and full optimization of ionic positions and volume and shape of the unit cell (ISIF = 3). For each, the force convergence was set to 10^–2^ eV/Å. To determine the energy versus volume relationships, we employed a method of interpolation and extrapolation between the structures from different phases for the ionic positions and volumes, as implemented in an in-house script.^46^ To obtain the energy of each structure corresponding to a specific volume, we optimized the structure with the volume held constant and both the ionic positions and cell shapes allowed to vary. To determine the minimum energy path between phases of CALF-20 (Figure S13), we employed the solid-state nudged elastic band (SS-NEB) technique,^47^ as integrated into the transition state atomistic simulation environment, which is an extension of the atomic simulation environment.^48^ The SS-NEB optimization was carried out using the FIRE algorithm. Helmholtz free energies were obtained from phonon partition functions obtained with the Phonopy software^49^ using the Parlinski-Li-Kawazoe method.^50^ To calculate phonons, we used a 2 × 2 × 2 supercell (to prevent interactions between periodic images) and reduced the *k*-point grid to 1 × 1 × 1 for all single-point finite displacement calculations. All calculated energies are provided per mol of unit cells, i.e. per formula containing 4 Zn cations.

Adsorption simulations were performed using the grand canonical Monte Carlo (GCMC) method, as implemented in RASPA software (version 2.0.47).^51^ The experimental structures of the γ and τ phases of CALF-20 from this study were employed. In these structures, the water molecules coordinating the Zn open metal sites lack hydrogen atoms and have partial occupancy (0.5). To address this in our simulations, every second water molecule in the Zn-oxalate chain was removed, and hydrogens were added. Their positions were optimized (with all other atoms’ coordinates being constrained) using geometry optimization in the Forcite module of Materials Studio, utilizing the universal force field.^52^ The Lennard-Jones and Coulomb potential parameters for the CO_2_ molecule were taken from the TraPPE model,^53^ while parameters for the MOFs were derived from the Dreiding force field.^54^ Partial charges for the MOFs were generated using PACMAN software,^55^ designed to reproduce DDEC6 partial charges.^56^ Electrostatic interactions were computed using Ewald summation (precision of 10^–6^). A cutoff of 14.0 Å was applied for both the Lennard-Jones potential and the real-space component of the Coulomb interactions. Tail correction was applied between adsorbate molecules but not for adsorbate–adsorbent interactions. The cross terms in the Lennard-Jones potential were determined using Lorentz–Berthelot mixing rules. The Peng–Robinson equation of state, with critical parameters (*T*_c_ = 303.67617 K, *p*_c_ = 7808379.87165 Pa, acentric factor = 0.24099), was used to convert pressure to fugacity and to account for the implicit bulk phase in the simulations.

The supercell used in the simulations consisted of 4 × 4 × 3 unit cells to satisfy the minimum image convention. The simulations were performed at 298 K. In the GCMC simulations, translation, rotation, reinsertion, and swap (insertion and deletion) moves were applied with equal probability. A total of 10^5^ Monte Carlo cycles were used for both the initialization and production phases. Each Monte Carlo cycle was defined as max (20, *N*) steps, where *N* represents the number of molecules in the simulation box.

## Results and Discussion

### Structural Characterization of a Novel γ-CALF-20 Phase

In previous reports on CALF-20, large variations were observed in the P-XRD patterns of the materials, depending on the history of the sample^16,23,28^ (Figures S1 and S2). For example, soaking CALF-20 in a 10% ammonia solution causes considerable changes in the reflection positions 100 and 011 (Figure S1). A similar effect was observed after 3 h of exposure to acidic gases (Figure S2). On the other hand, we found that the powder pattern of raw bulk CALF-20 did not match the simulated pattern (Figure S3). A similar observation was made by Wei et al.^28^ regarding the mismatch between the simulated patterns of CALF-20 and those recorded for as-synthesized and activated materials (Figure S1). Considering the observed structural changes, we assume that there exists a novel phase of CALF-20.

**Figure 1:**
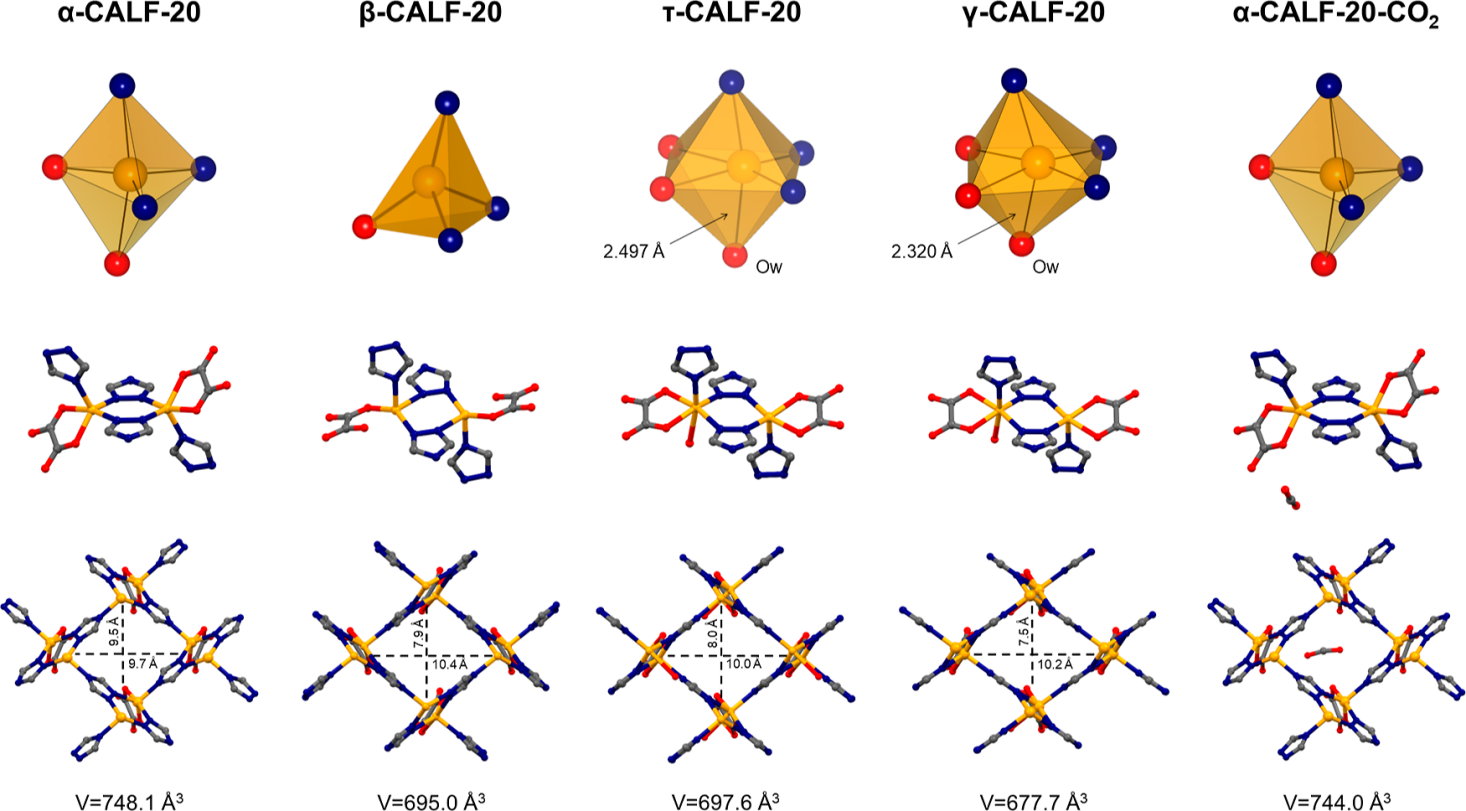
Experimental insight into the transformability of CALF-20: geometries of zinc cation coordination spheres (top row), coordination environments of zinc cations (middle row), and views of the 3-D frameworks along the *x* axis (bottom row) of α-CALF-20 ([Zn_2_(ox)(trz)_2_]·s; s—solvent) as-synthesized, β-CALF-20 ([Zn_2_(ox)(trz)_2_]) reported by Chen et al.^23^ formed after exposing bulk α-CALF-20 to over 23% relative humidity, τ-CALF-20 ([Zn_2_(ox)(trz)_2_(H_2_O)]·s) obtained by heating α-CALF-20 at 80 °C for 7 days, γ-CALF-20 ([Zn_2_(ox)(trz)_2_(H_2_O)]·s) obtained by heating α-CALF-20 at 80 °C for 10 days, and α-CALF-20-CO_2_ ([Zn_2_(ox)(trz)_2_]·CO_2_) resulting from exposure of activated α-CALF-20 to CO_2_ at 10 bar. Color code: Zn–yellow, C—gray, O—red, N—blue; O_w_—oxygen atom from water molecule coordinated to Zn. Hydrogen atoms have been omitted for clarity. Note that all structures were determined from single crystals, except for β-CALF-20, which was obtained from powder.

**Figure 2:**
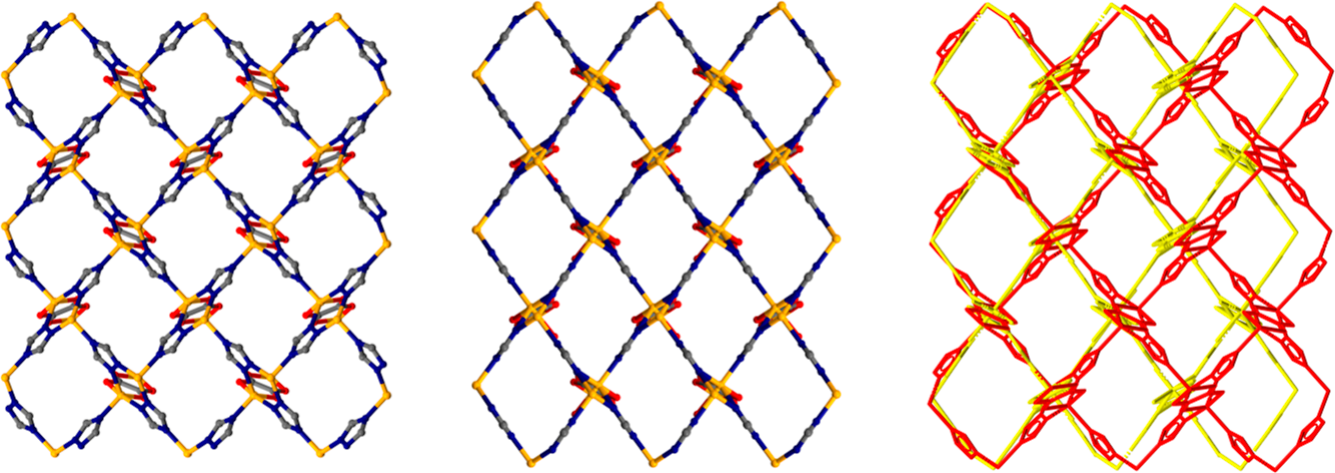
Views of α-CALF-20 (left), γ-CALF-20 (center), and an overlay of these structures (right) along the *x* axis. Hydrogen atoms have been omitted for clarity.

To test this hypothesis, we identified suitable conditions for the CALF-20 single-crystal-to-single-crystal transformation. First, we obtained crystals using the reported crystallization method.^16^ During crystallization, 2,5-dihydroxybenzoquinone is hydrolyzed to the oxalate anion(ox), which, in the presence of zinc(II) and triazolate (trz), forms the first-known crystalline phase of CALF-20, Zn_2_(ox)(trz)_2_ s (s = solvent molecules), hereafter referred to as α-CALF-20 (Figures 1 and S4). The ASU of α-CALF-20 contains a zinc ion, a triazolate ligand, and half of an oxalate ligand. Each Zn^2+^ center is five-coordinated to three triazolate ions and one oxalate ion, resulting in a distorted trigonal bipyramidal geometry. The nitrogen atoms at the 1,2 positions of the trz ligand link the nearest two zinc centers into a planar Zn–N–N–Zn–N–N binuclear ring. Adjacent units are then connected by the remaining nitrogen atoms so that their planes form a dihedral angle of 65.75° (Figure S5). Such an arrangement leads to the formation of corrugated layers, which are linked by the oxalate ligands into a three-dimensional network exhibiting monoclinic symmetry and dmc topology (Figures 2, S6 and S7). A two-dimensional set of channels of 3.577 by 4.153 Å and 5.961 by 5.570 Å observed in the lattice along [100] and [001], respectively, comprises 39.4% of the unit cell volume as calculated with a probe radius of 1.3 Å (Figure S8).

Interestingly, heating α-CALF-20 crystals exposed to air at 80 °C for 10 days triggered a single-crystal-to-single-crystal transformation. The new phase named γ-CALF-20 ([Zn_2_(ox)(trz)_2_(H_2_O)·s]; Figures 1 and S4), although adopting the same monoclinic space group *P*2_1_/*c* as its predecessor α-CALF-20 (Figure S7), exhibits significant changes in the unit cell parameters and volume. Comparison of the crystal structures reveals lengthening of the crystallographic axes *a* from 8.9735(2) to 9.3919(5) Å and *c* from 9.5429(2) to 10.2078(6) Å, with concomitant shortening of the *b* axis from 9.7321(1) to 7.5264(4) Å upon proceeding from α-CALF-20 to γ-CALF-20. There is also a decrease in the crystallographic β angle from 115.6790(10) to 110.078(6)° and in volume from 748.07(3) to 677.71(7) Å^3^ (Table S1). However, the most important difference between α-CALF-20 and γ-CALF-20 is evident in the coordination spheres of the Zn^2+^ centers. In γ-CALF-20, the zinc coordination sphere is expanded to include a water molecule (Figures 1 and S4), which, interestingly, is statistically ligated to only half of the Zn^2+^ ions in the MOF structure (the occupancy factor for H_2_O was refined to 0.5). This corresponds to a change in the coordination number (CN) from 5 to 6 for half of the zinc cations in the lattice, resulting in the presence of two different coordination sphere geometries in the same γ-CALF-20 crystal: half of the zinc cations exhibit a distorted square pyramidal geometry (CN = 5), while the others have a distorted octahedral geometry (CN = 6) (Figure S9). It is worth noting that in both CALF-20 phases there are zinc centers that have a CN of 5, but the type of geometry is different: in α-CALF-20 the Zn^2+^ ions adopt a distorted trigonal bipyramidal geometry, while in γ-CALF-20 a distorted square pyramidal geometry is observed (Tables S2 and S3). The spatial arrangement of the Zn–N–N–Zn–N–N binuclear units in γ-CALF-20 deviates from the planar distribution observed in α-CALF-20, which is evidenced by torsion angles ranging from ±16.26–20.47° in γ-CALF-20 vs ±0.54–0.66° in α-CALF-20 (Figure S5). In γ-CALF-20, the binuclear rings are mutually oriented in a nearly perpendicular manner; the angle between the planes of adjacent rings is 89.59°, whereas in α-CALF-20 they are arranged obliquely at an angle of 65.75° (Figure S5). The corrugated Zn-trz layers joined by the oxalate ions constitute the three-dimensional dmc network as in α-CALF-20 (Figures 2, S6 and S7). The guest-accessible space in γ-CALF-20 corresponds to an alternating arrangement of 1D channels of 2.389 by 3.225 Å and stacks of isolated voids that both propagate along [100]. The total void volume, calculated using a probe radius of 1.3 Å, is 11.7% (Figure S8).

We found that heating α-CALF-20 at 80 °C for only 7 days allowed us to observe another CALF-20 phase. Its structure can be considered an intermediate between α-CALF-20 and γ-CALF-20; therefore, we named it τ-CALF-20. This phase exhibits the same symmetry as γ-CALF-20, with only minor differences in unit cell parameters (Table S1) and displays a similar geometry of the Zn^2+^ coordination sphere. However, the Zn–O_w_ distance in τ-CALF-20 is longer than in γ-CALF-20, measuring 2.497 Å instead of 2.320 Å (see Tables S3 and S4), thus showing how a water molecule approaches the metallic center. We noted that both bulk α-CALF-20 and bulk γ-CALF-20 spontaneously transform into τ-CALF-20 after a certain period of time, while attempts to reverse this transformation have been unsuccessful so far. These observations demonstrate that τ-CALF-20 appears to be the most stable of all phases investigated.

The structures of the two new CALF-20 phases reported in this paper differ considerably from the β-CALF-20 phase recently identified from powder by Chen et al.^23^ (Figure S6, Table S5). This material was obtained when bulk α-CALF-20 was exposed to relative humidity of over 23% for 3 days at room temperature. The differences among the β-, τ-, and γ-CALF-20 phases are particularly noticeable in the coordination spheres of the zinc ions. Compared to the five-coordinated Zn^2+^ center found in α-CALF-20, the zinc ions in β-CALF-20 are four-coordinated. This change in CN is attributed to the elongation and subsequent breakage of a Zn–O bond between the metal and the oxalate ligand (Figure S6). On the other hand, τ-CALF-20 and γ-CALF-20 show an increase in CN from 5 to 6 due to the inclusion of a water molecule in the coordination sphere of the zinc ion, as discussed previously.

**Figure 3:**
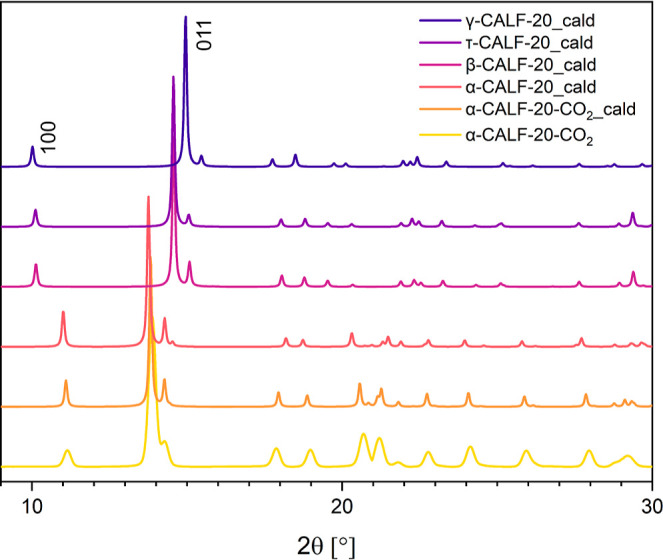
Comparison of P-XRD patterns of all studied phases of CALF-20 (from top to bottom): calculated γ-CALF-20, calculated τ-CALF-20, calculated β-CALF-20 reported by Chen et al.,^23^ calculated α-CALF-20, calculated α-CALF-20-CO_2_, and experimental α-CALF-20-CO_2_.

Moreover, the comparison of P-XRD patterns of CALF-20 phases provides compelling proof of their distinct nature. The α-CALF-20 → γ-CALF-20 phase transformation can be clearly observed in Figure 3, where the reflection positions of 100 and 011 experience a shift from 11.0 to 10.0° and from 13.8 to 15.0°, respectively. Significant differences in powder patterns also appear between the γ-CALF-20 and β-CALF-20 phases, thus confirming that γ-CALF-20 is a newly discovered phase. One noticeable distinction lies in the disparity in the position of reflection 011. In γ-CALF-20, the reflection is detected at a 2θ angle of 15.0°, whereas in β-CALF-20, the reflection occurs at a 2θ angle of 14.5° (Figure 3). Notably, the P-XRD pattern generated from the single crystal structure of τ-CALF-20 aligns perfectly with the established β-CALF-20 pattern generated from powder data found in existing literature.^23^ However, despite this alignment, there are notable disparities in their respective structures, as discussed earlier. As the authors of that mentioned report write, “the reduction of the metal CN, with the Zn changing from five- to four-coordinated, in the presence of water is highly unusual”.^23^ Furthermore, given the unusual geometry of the β-CALF-20 framework, it may be of interest to revisit some of the details of the structure derived from the powder data.

### In Situ SC-XRD Analysis of CO_2_ Binding Sites

**Figure 4:**
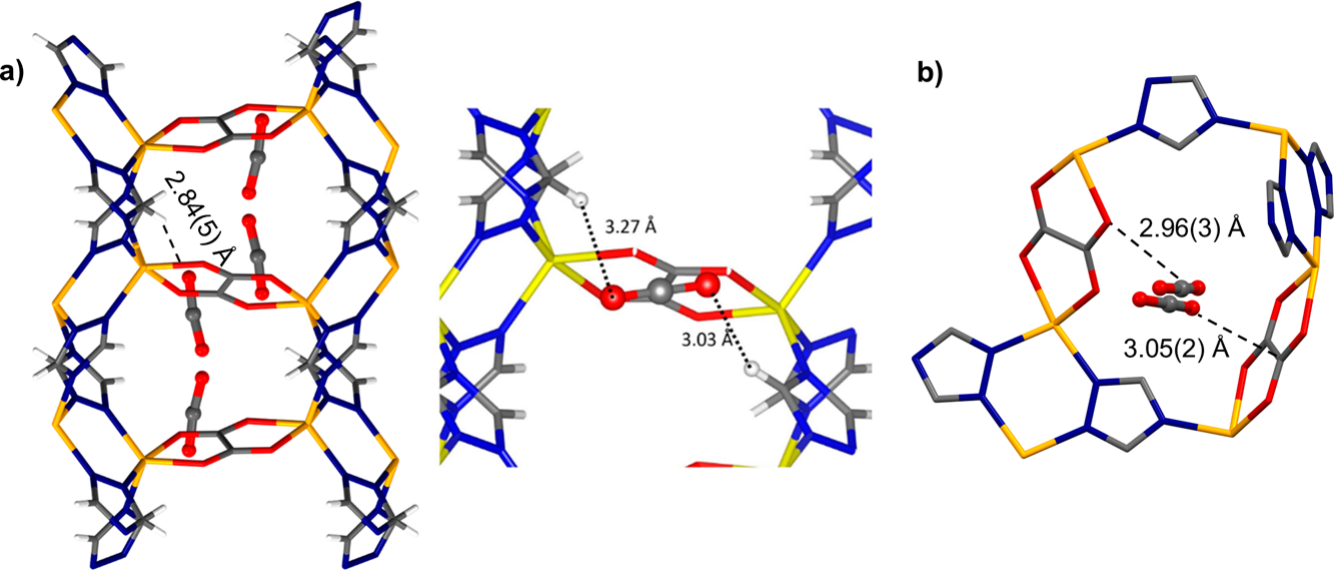
(a) Experimentally determined CO_2_ binding sites in α-CALF-20-CO_2_ (left) compared to the theoretical binding sites calculated by Lin et al. (right) viewed along the *c* axis. From Jian-Bin Lin *et al.*, A scalable MOF as a durable physisorbent for carbon dioxide capture. *Science 374*, 1464–1469 (**2021**). DOI:10.1126/science.abi728116. Reprinted with permission from AAAS. (b) The two shortest interatomic distances between CO_2_ and the oxalate ion in α-CALF-20-CO_2_. Hydrogen atoms have been omitted for clarity.

Lin et al.^16^ performed simulations to identify likely CO_2_ binding sites in CALF-20. To verify their findings, we carried out in situ SC-XRD studies under controlled CO_2_ gas pressure using an environmental gas cell.^36^ A single crystal of α-CALF-20 was subjected to vacuum at the temperature of 80 °C for 3 h in order to remove residual solvent molecules from the lattice. Activated α-CALF-20-act was then exposed to CO_2_ at 10 bar for 1 h at room temperature, thus forming [Zn_2_(ox)(trz)_2_]·CO_2_ (α-CALF-20-CO_2_; Figures 1 and S4). Subsequent SC-XRD revealed that the framework underwent a slight expansion from 9.7321(1) to 9.8435(3) Å along the [010] direction and a contraction from 9.5429(2) to 9.4623(3) Å in the [001] direction during CO_2_ sorption (Figure S10, Table S1), which is consistent with recent theoretical calculations made by Fan et al.^25^ The difference in the electron count before and after CO_2_ exposure, calculated using Olex,^57^ is 10 per ASU cell and correlates well with the occupancy estimated during the structure refinement, i.e., 0.45 CO_2_ molecules per ASU. The CO_2_ molecule is disordered over two symmetry-related positions, refining to site occupancies of 0.25 for each disordered component (Figure 4). None of these positions appear to be consistent with the calculations reported by Lin et al.^16^ While those authors estimated that the CO_2_ is aligned parallel to the oxalate moiety, with the shortest distance of 3.03 Å between oxygen atom of the CO_2_ and the hydrogen atom of the triazolate, we found that the same distance in α-CALF-20-CO_2_ is 2.84(5) Å, and the orientation of CO_2_ in relation to the oxalate fragment is nearly perpendicular (Figure 4). The oxygen atom of the CO_2_ molecule is positioned near the carbon atom of the oxalate ion with the distance between these atoms measuring 3.05(2) Å. However, the distance between the oxalate oxygen atom and the CO_2_ carbon atom equals 2.96(3) Å. The mutual orientation of carbon dioxide and the oxalate moiety, along with the fact that both groups have a partial positive charge on the carbon atoms and a negative charge on the oxygen atoms, indicates that the interaction between CO_2_ and CALF-20 framework in the crystals is influenced by electrostatic forces (Figure 4).

We compared our results to the adsorption data reported by Lin et al.^16^ In this work, an in situ CO_2_ diffraction experiment on the single crystal was performed under a pressure of 10 bar, whereas the CO_2_ sorption isotherm as reported by in the above-mentioned report^16^ only reached a maximum pressure of 1.2 bar. Therefore, for a direct comparison of the CO_2_ loadings between our results and the reported data, we utilized the Sips equation *n* = (*n*_*m*_(Kp)^*m*^)/(1 + (Kp)^*m*^) and the Excel Solver add-in to determine the constants *n*_*m*_, K, and m that most accurately matched the literature data. This enabled us to calculate *n*(fit) for a wider pressure range. Through this procedure, we estimated that the maximum CO_2_ uptake for α-CALF-20 at 10 bar and 293 K is 5 mmol/g (Figure S11), which corresponds to approximately 0.89 CO_2_ molecules per ASU. This value is greater than 0.45 CO_2_ molecule found in the crystal structure, which suggests that the α-CALF-20 single crystal may not have been fully saturated with carbon dioxide during the sorption experiment. We suspect that this is due to a partial blockage of the pores caused by the residual solvent molecules. After the activation process, the electron count decreased to 10 e̅/ASU and further removal of the solvent resulted in the destruction of the crystal. This leads us to the conclusion that a full activation of CALF-20 is only possible for the bulk material, but not for single crystals.

In addition, we exposed the activated α-CALF-20 powder to CO_2_ and subsequently recorded its P-XRD pattern. Comparing the experimental powder pattern for α-CALF-20-CO_2_ with the simulated one indicates that the behavior of the postadsorption material corresponds to that observed in the single crystal (Figure 3).

### Computational Studies on CALF-20 Phases

**Figure 5:**
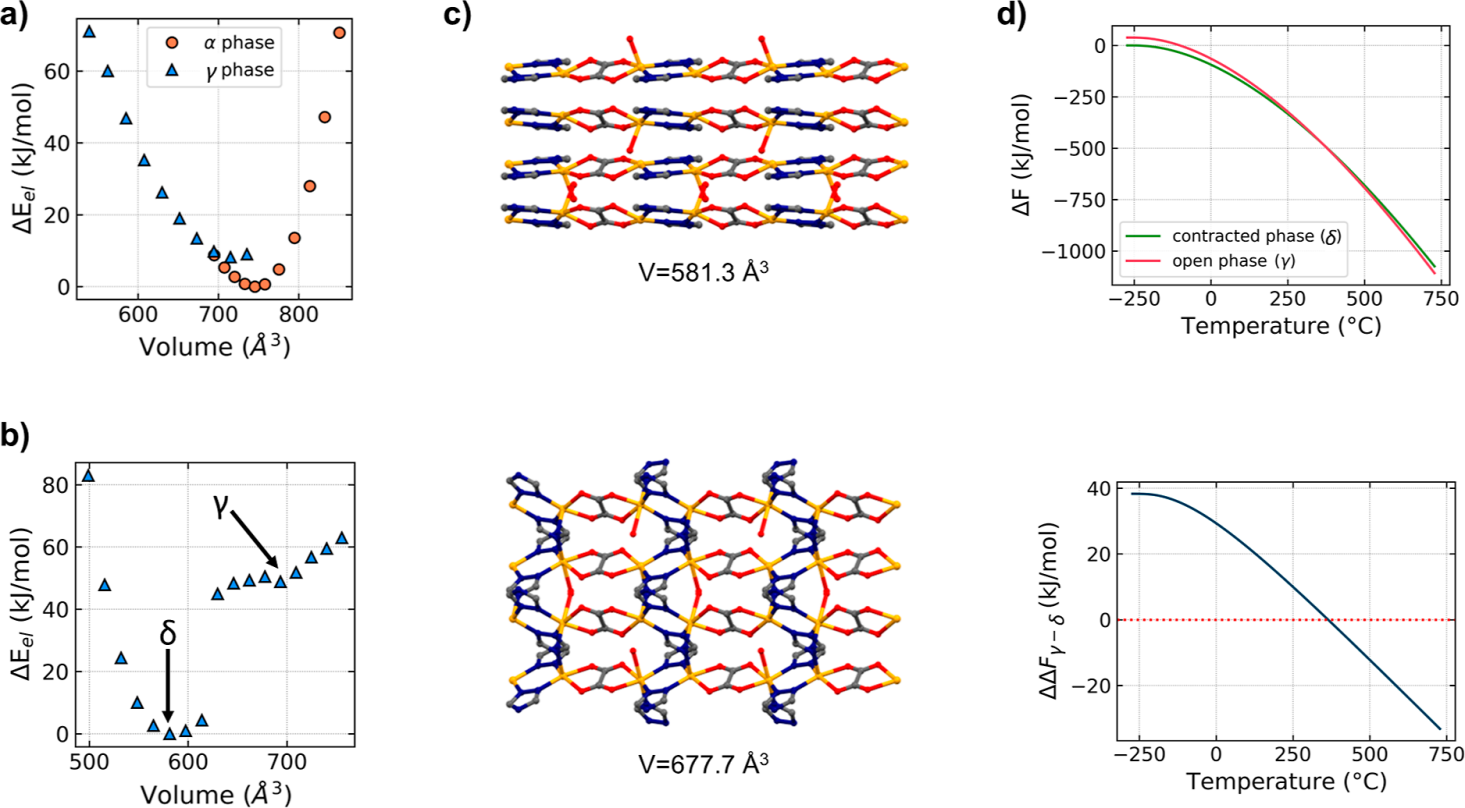
Energy–volume dependence of different phases of CALF-20 in (a) the absence and (b) the presence of water molecules. In the absence of water, the α phase is stable. In the presence of water, the δ phase is stable, whereas the γ phase shows metastability. Note that the energies do not consider any temperature contribution; see text for details. (c) Comparison between the hypothetical contracted (δ-CALF-20; top) and experimental (γ-CALF-20; bottom) phases of CALF-20 viewed along the *z* axis (hydrogen atoms omitted for clarity), and (d) the free energies of these phases.

To better understand the nature of the transformation of α-CALF-20 into γ-CALF-20, we employed DFT. Initially, we optimized the positions of the ions in the α and γ CALF-20 phases without changing the experimental unit cell parameters. The relative stability was calculated in both the presence and absence of water molecules in the unit cell. Under dry conditions, α-CALF-20 was found to be 12.0 kJ/mol (mole of unit cells, stoichiometrically equivalent to 4 Zn sites) more stable than γ-CALF-20, corroborating experimental observations. However, when four water molecules are introduced into the system (one molecule per Zn^2+^ center), γ-CALF-20 demonstrates a slight stability advantage of 6.5 kJ/mol, with water binding to the exposed Zn site. These minor energy differences imply that the material could be susceptible to structural deformations under various conditions. To investigate this, we analyzed the energy-volume relationship by optimizing atomic positions and cell shapes over a range of constrained volumes. In a dry environment, both phases exhibit a typical parabolic *E* vs *V* dependence, with α-CALF-20 being more stable by 8.2 kJ/mol than the metastable anhydrous γ-CALF-20 (Figure 5a).

When the unit cell volume of anhydrous γ-CALF-20 exceeds 735 Å^3^, it becomes unstable, causing the optimization to converge toward α-CALF-20. To determine whether the transition between the two phases under dry conditions occurs spontaneously, we performed a SS-NEB analysis of the transformation path. The results depicted in Figure S12 reveal no transition state. Considering the lower stability of anhydrous γ-CALF-20 and the absence of a significant transformation barrier, the calculations suggest that the α phase is the only observable phase under dry conditions, in agreement with experimental results (this work and ref (23)).

The energy-volume relationship becomes more complicated in the presence of water (Figure 5b). First, the full, unconstrained geometry optimization (ionic position and volume) leads to contracted nonporous phase, δ-CALF-20 (Figures 5c and S13), which is more stable than the known phase, γ-CALF-20, by approximately 50 kJ/mol (electronic energy). Interestingly, calculations described in a previous report by Oktavian et al.^20^ indicate a possible existence of a closed pore phase derived from α-CALF-20 with a unit cell volume predicted to measure 585 Å^3^. This value is strikingly similar to the unit cell volume of δ-CALF-20 which is 581.3 Å^3^. δ-CALF-20 contains water molecules bonded to zinc cations and forming hydrogen bonds with the oxygen from the oxalate linker. Additionally, due to the relaxation of the strain in the system and strong interaction between unsaturated nitrogen and H_H2O_, the Zn–N bond involving the trz linker opposite to the bound water breaks. The transition between γ-CALF-20 and δ-CALF-20 occurs around 625 Å^3^ as a step-change with an energy difference of 40 kJ/mol (Figure 5b). Since δ-CALF-20 was not observed under experimental conditions, and we only considered the electronic energy in these calculations, we would expect that it must be stable only at very low temperatures. To evaluate this hypothesis, we calculated Helmholtz free energies as a function of temperature for δ-CALF-20 (581.3 Å^3^) and γ-CALF-20 (677.7 Å^3^). Figure 5d shows that the stability of these phases may change as the temperature increases. As expected, at lower temperatures δ-CALF-20 can be considered as stable, while at higher temperatures, the increased entropy stabilizes γ-CALF-20. The transition temperature is predicted to be 360 °C. This overestimation is most likely related to the limitation of the harmonic approximation used to calculate the vibrational enthalpy and entropy from phonon frequencies and known overestimation^58^ of dispersion forces by empirical dispersion correction. Considering anharmonicity in a system as large as a MOF is currently not possible due to significant computational cost. Moreover, an overestimation of the transition temperature has previously been reported for MIL-53.^59^ Hence, this computational observation should be considered as more qualitative than quantitative, and it suggests possible contraction of hydrated CALF-20 at very low temperatures. Furthermore, considering the magnitude of the structural changes, including framework dimensionality reduction from 3D to 2D and coordination bond breaking, high pressure might be necessary to induce the γ-CALF-20 → δ-CALF-20 transformation.

Furthermore, we have theoretically investigated the adsorption properties of τ-CALF-20 and γ-CALF-20 toward CO_2_. Using GCMC simulations, we examined two distinct cases. In the first one, all water molecules bound to zinc cations were artificially removed, whereas in the second scenario, water was coordinated to the metal centers with a fixed H_2_O/Zn ratio of 1:2, as determined by SC-XRD experiments. Subsequently, theoretical CO_2_ adsorption isotherms were compared to that of α-CALF-20 (Figure S14). Notably, the CO_2_ uptake trends remained consistent for each phase, regardless of whether coordinated water molecules were included in the model.

Remarkably, γ-CALF-20 predominantly shows nonporous characteristics with regard to CO_2_. The maximum CO_2_ uptake for this phase oscillates around 0.5 mmol/g at 1 bar. Conversely, τ-CALF-20 exhibits a significantly enhanced capacity for carbon dioxide adsorption, with the maximum uptake exceeding 2.5 mmol/g at 1 bar CO_2_ pressure. Nevertheless, this value is still significantly lower than that of α-CALF-20, which reaches a maximum uptake of 4 mmol/g at 1 bar.

## Conclusions

Our study has yielded several significant findings regarding CALF-20. We have identified and structurally characterized two novel γ-CALF-20 and τ-CALF-20 phases, which can be obtained after heating the known α-CALF-20 phase at 80 °C in an open vessel. Compared to α-CALF-20, the Zn centers in τ- and γ-CALF-20 have a different coordination environment, with one coordinated water molecule. Furthermore, we predict the existence of an additional dense phase, δ-CALF-20. This contracted phase may only exist at very low temperatures or, more likely, under high pressure. As part of our research, we monitored the CO_2_ sorption process of CALF-20 using a single-crystal X-ray environmental gas cell. We found that CO_2_ sorption in α-CALF-20 induces slight structural changes. Detailed analysis of α-CALF-20-CO_2_ revealed that electrostatic interactions may play an important role in carbon dioxide binding. Overall, our findings significantly improve the understanding of CALF-20 chemistry and have the potential to facilitate further advances in carbon capture and storage technologies. A better understanding of the structural changes in CALF-20 will allow for further optimization of its use on an industrial scale and for mastering any changes that occur during the process.

## Supporting Information Available

Additional figures depicting all crystal structures, powder patterns, IR spectra, a graph depicting results of theoretical calculations, crystallographic data for all crystals described (PDF)
