# Supplementary material for: Unveiling Temperature-Induced Structural Phase Transformations and CO2 Binding Sites in CALF-20
Source: Inorg Chem. 2024 Sep 27;63(41):19277–86. doi: 10.1021/acs.inorgchem.4c02952 (PMC11483831; doi:10.1021/acs.inorgchem.4c02952)
Supplement: Supplementary file 1 — ic4c02952_si_001.pdf [file ic4c02952_si_001.pdf]

SUPPLEMENTARY INFORMATION FILE ACCOMPANYING:

Unveiling Temperature-Induced Structural Phase Transformations and CO_2 Binding Sites in CALF-20

Joanna Drwęska[a], Filip Formalik[b,c], Kornel Roztocki[a], Randall Q. Snurr[c], Leonard J. Barbour[d], and Agnieszka Janiak[a]**

[a] Faculty of Chemistry, Adam Mickiewicz University, Uniwersytetu Poznańskiego 8, 61-614 Poznań, Poland
[b] Department of Micro, Nano, and Bioprocess Engineering, Faculty of Chemistry, Wroclaw University of Science and Technology, Wybrzeże Wyspiańskiego 27, 50-370 Wrocław, Poland
[c] Department of Chemical and Biological Engineering, Northwestern University, Evanston, Illinois, 60208, United States
[d] Department of Chemistry and Polymer Science, Stellenbosch University, Private Bag X1, Matieland 7602, South Africa

**e-mail addresses: agnieszk@amu.edu.pl, kornel.roztocki@amu.edu.pl*

Table of Contents

Figures S3
Tables S15

Figures

Figure S1 Previously published P-XRD patterns of activated (CALF-20), air-dried (CALF-20-Raw), and simulated (CALF-20-Sim.) CALF-20 framework (left) and activated CALF-20 treated by different solutions (right; B – base solution, W – water, MA – moist air followed by another activation, A – acid solution) simulated and measured by Wei et al.[1] The samples were activated under vacuum at 333–373 K. Reproduced from Ref. 1 with permission from the Royal Society of Chemistry. S3
Figure S2 Previously published P-XRD patterns of pristine CALF-20 and CALF-20 after three-hour exposure to acid gases as listed. From Jian-Bin Lin et al., A scalable metal-organic framework as a durable physisorbent for carbon dioxide capture. Science 374, 1464-1469 (2021). DOI:10.1126/science.abi7281[2]. Reprinted with permission from AAAS. S3
Figure S3 Calculated P-XRD patterns of published CALF-20 (CSD refcode TASYAR)[2], α-CALF-20, β-CALF-20 reported by Chen et al.[3], τ-CALF-20, γ-CALF-20, and an experimental pattern of raw bulk CALF-20 which indicates that the crude product of bulk synthesis reported by Lin et al.[2] is not α-CALF-20, but γ-CALF-20. .... S4
Figure S4 Comparison of asymmetric units of all known phases of CALF-20. .... S4
Figure S5 Views of Zn-trz corrugated layers (top row) and torsion angles within the Zn-N-N-Zn-N-N units (bottom row) in α-CALF-20 (left) and γ-CALF-20 (right). Hydrogen atoms have been omitted for clarity. S5
Figure S6 Views of α-CALF-20, β-CALF-20 reported by Chen et al.,[3] τ-CALF-20, and γ-CALF-20 along the x, y, and z axes, and an overlay of these structures. Hydrogen atoms have been omitted for clarity. . S6
Figure S7 Simplified views of α-CALF-20 (left) and γ-CALF-20 (right) along the y axis depicting their **dmc** topology. The zinc cations are simplified into a 4-connected node (yellow) whereas the triazolate ligands are represented as a 3-connected node (blue). Water molecules coordinated to Zn^{2+} centers in γ-CALF-20 are shown in red. S7

Figure S8 Solvent-accessible voids in α-CALF-20 (left) and γ-CALF-20 (right) calculated with a probe radius of 1.3 Å viewed along the x axis. Hydrogen atoms have been omitted for clarity. S7
Figure S9 Comparison of coordination spheres of Zn^{2+} centers simultaneously present in γ-CALF-20: the five-coordinated (left) and the six-coordinated (right) containing a water molecule.................................S8
Figure S10 Overlay of α-CALF-20 (red), α-CALF-20-act (yellow), and α-CALF-20-CO_2 (blue) structures in a view along the x (top), y (center), and z (bottom) axes. S9
Figure S11 The extrapolated CO_2 adsorption isotherm based on the data reported by Lin et al.[2] S10
Figure S12 SS-NEB analysis of the transformation between α-CALF-20 and γ-CALF-20 showing no transition state without the presence of water molecules in the pores. S10
Figure S13 a) The predicted crystal structure of δ-CALF-20, b) a chain formed by zinc cations coordinated to triazolate and oxalate, c) view of the crystal structure along the y axis. Hydrogen atoms have been omitted for clarity. S11
Figure S14 CO_2 adsorption isotherms for α-CALF-20 (determined experimentally by Lin et al.[2]), τ-CALF-20, and γ-CALF-20 (determined theoretically for both phases without the presence of coordinated water molecules $[Zn_2(ox)(trz)_2]$ [dry] and with water included in the model $[Zn_2(ox)(trz)_2(H_2O)]$ [wet]). S11
Figure S15 P-XRD patterns of synthesized and calculated zinc oxalate dihydrate. S12
Figure S16 FT-IR spectra of three CALF-20 phases. S12
Figure S17 The thermogravimetric analysis (blue) and differential scanning calorimetry (red) curves of bulk γ-CALF-20...S13
Figure S18 Experimental powder patterns of τ-CALF-20 after heating at 150 °C under vacuum for four hours (left) and heating at 80 °C in air for twelve days (right). S13
Figure S19 Difference (Fo-Fc) electron density map for α-CALF-20-CO_2. ....S14

Tables

Table S1 Crystallographic data for α-CALF-20, γ-CALF-20, τ-CALF-20, α-CALF-20-act, and α-CALF-20-CO_2. ....S15
Table S2 Selected bond distances [Å] and angles [°] for α-CALF-20. ....S16
Table S3 Selected bond distances [Å] and angles [°] for γ-CALF-20. ....S16
Table S4 Selected bond distances [Å] and angles [°] for τ-CALF-20..S16
Table S5 Comparison of unit cell parameters of α-CALF-20, β-CALF-20 reported by Chen et al.[3], τ-CALF-20, and γ-CALF-20...S17
Table S6 Unit cell parameters of α-CALF-20, γ-CALF-20, and δ-CALF-20 obtained from the DFT calculations. S17

Figures

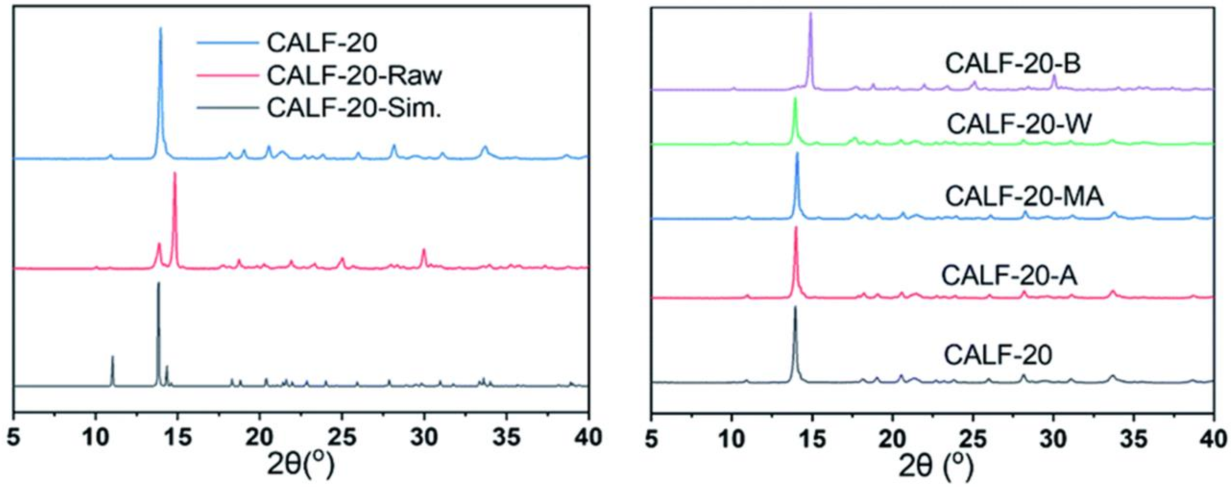

***Figure S1** Previously published P-XRD patterns of activated (CALF-20), air-dried (CALF-20-Raw), and simulated (CALF-20-Sim.) CALF-20 framework (left) and activated CALF-20 treated by different solutions (right; B – base solution, W – water, MA – moist air followed by another activation, A – acid solution) simulated and measured by Wei* et al.[1] *The samples were activated under vacuum at 333–373 K. Reproduced from Ref. 1 with permission from the Royal Society of Chemistry.*

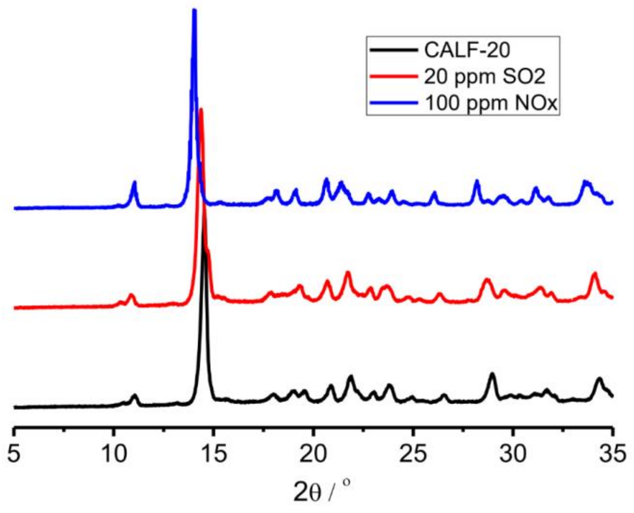

***Figure S2** Previously published P-XRD patterns of pristine CALF-20 and CALF-20 after three-hour exposure to acid gases as listed. From Jian-Bin Lin et al., A scalable metal-organic framework as a durable physisorbent for carbon dioxide capture. Science 374, 1464-1469 (2021). DOI:10.1126/science.*abi7281[2]. Reprinted with permission from AAAS.

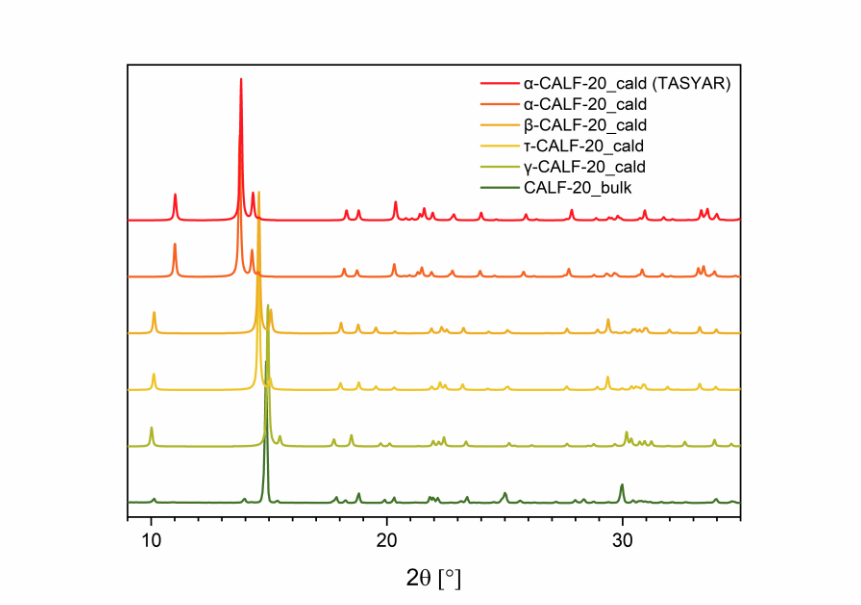

Figure S3 *Calculated P-XRD patterns of published CALF-20 (CSD refcode TASYAR)*[2]*, α-CALF-20, β-CALF-20 reported by Chen* et al.[3]*, τ-CALF-20, γ-CALF-20, and an experimental pattern of raw bulk CALF-20 which indicates that the crude product of bulk synthesis reported by Lin et al.*[2] *is not α-CALF-20, but γ-CALF-20.*

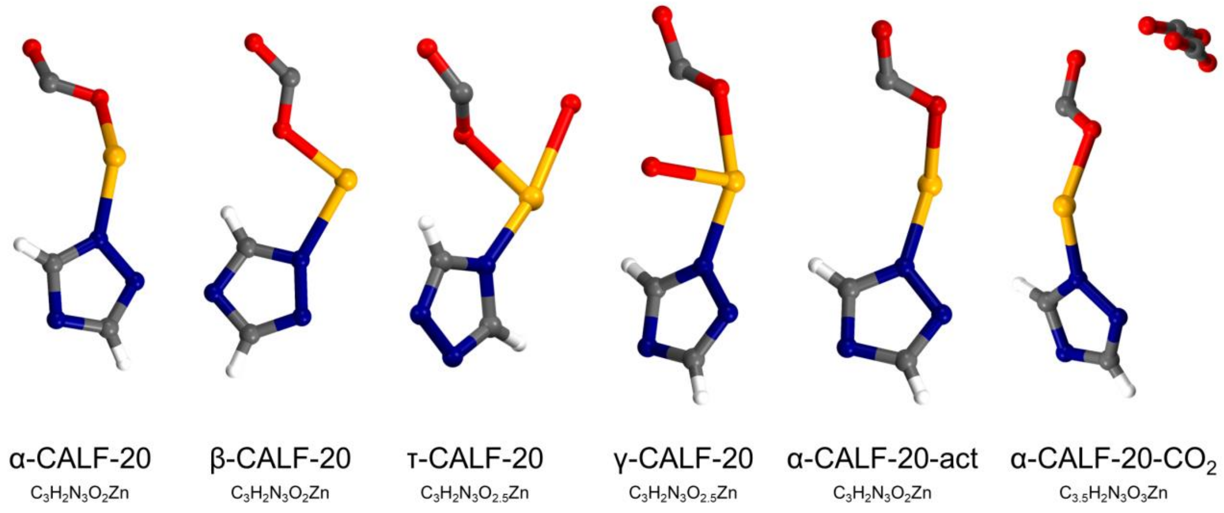

Figure S4 *Comparison of asymmetric units of all known phases of CALF-20.*

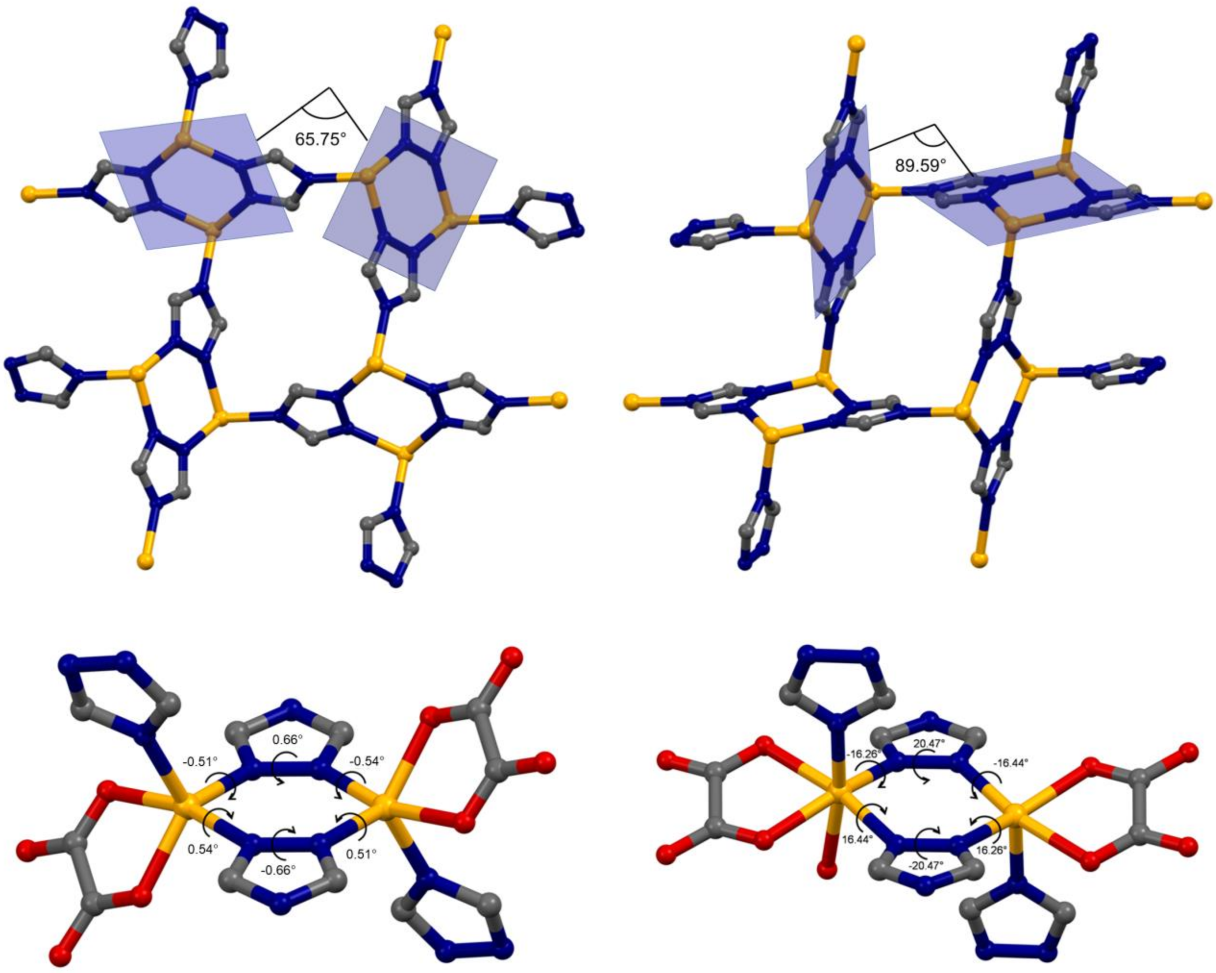

Figure S5 *Views of Zn-trz corrugated layers (top row) and torsion angles within the Zn-N-N-Zn-N-N units (bottom row) in α-CALF-20 (left) and γ-CALF-20 (right). Hydrogen atoms have been omitted for clarity.*

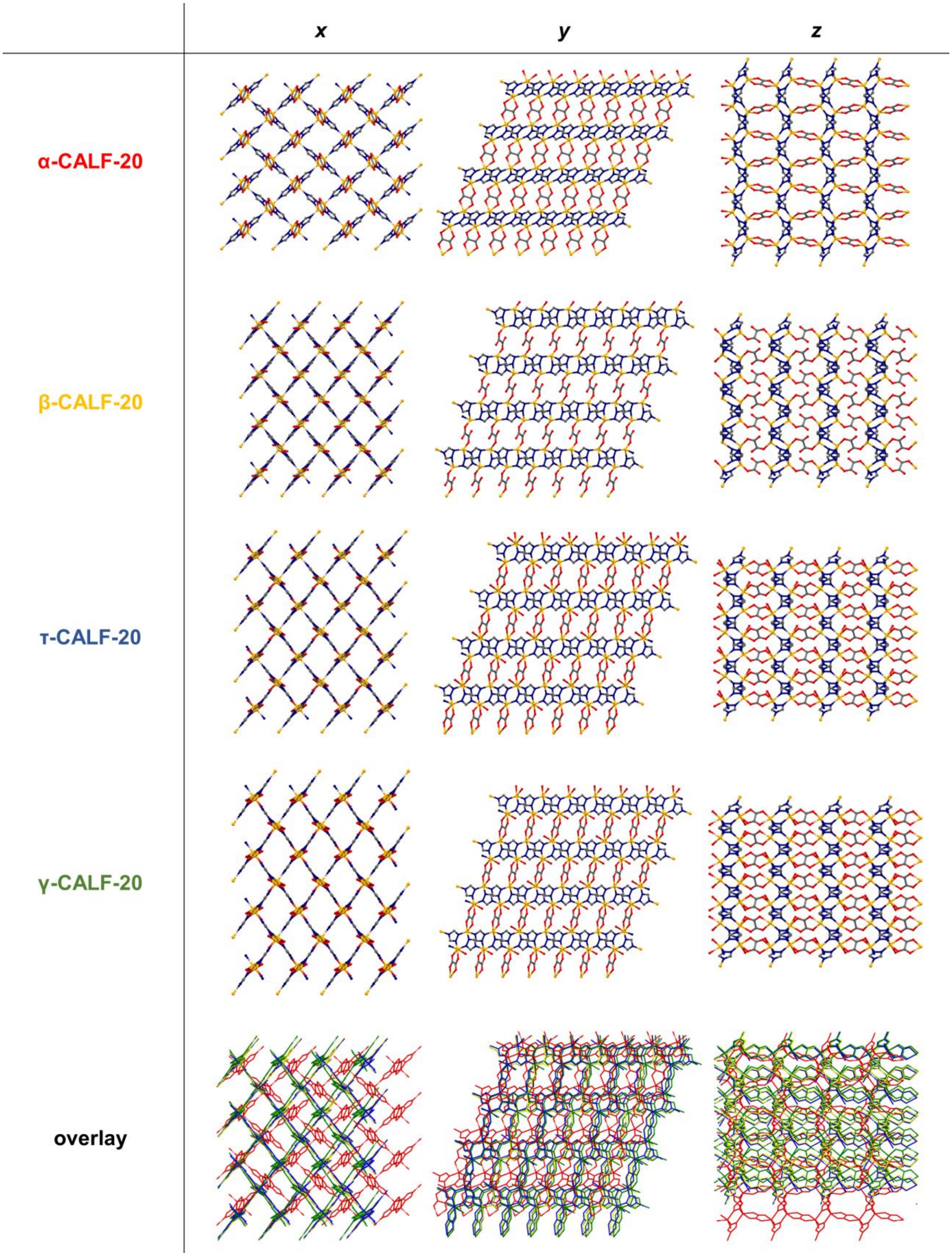

Figure S6 *Views of α-CALF-20, β-CALF-20 reported by Chen et al.,*[3] *τ-CALF-20, and γ-CALF-20 along the* x, y, *and* z *axes, and an overlay of these structures. Hydrogen atoms have been omitted for clarity.*

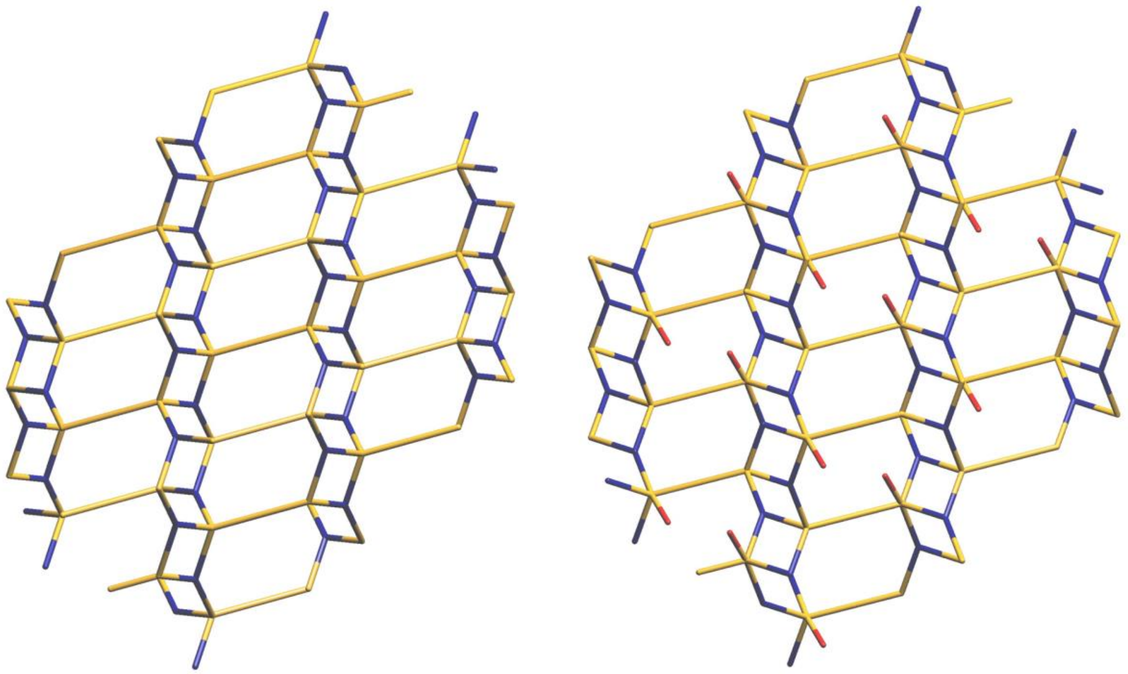

Figure S7 *Simplified views of α-CALF-20 (left) and γ-CALF-20 (right) along the* y *axis depicting their* ***dmc*** *topology. The zinc cations are simplified into a 4-connected node (yellow) whereas the triazolate ligands are represented as a 3-connected node (blue). Water molecules coordinated to* Zn^{2+} *centers in γ-CALF-20 are shown in red.*

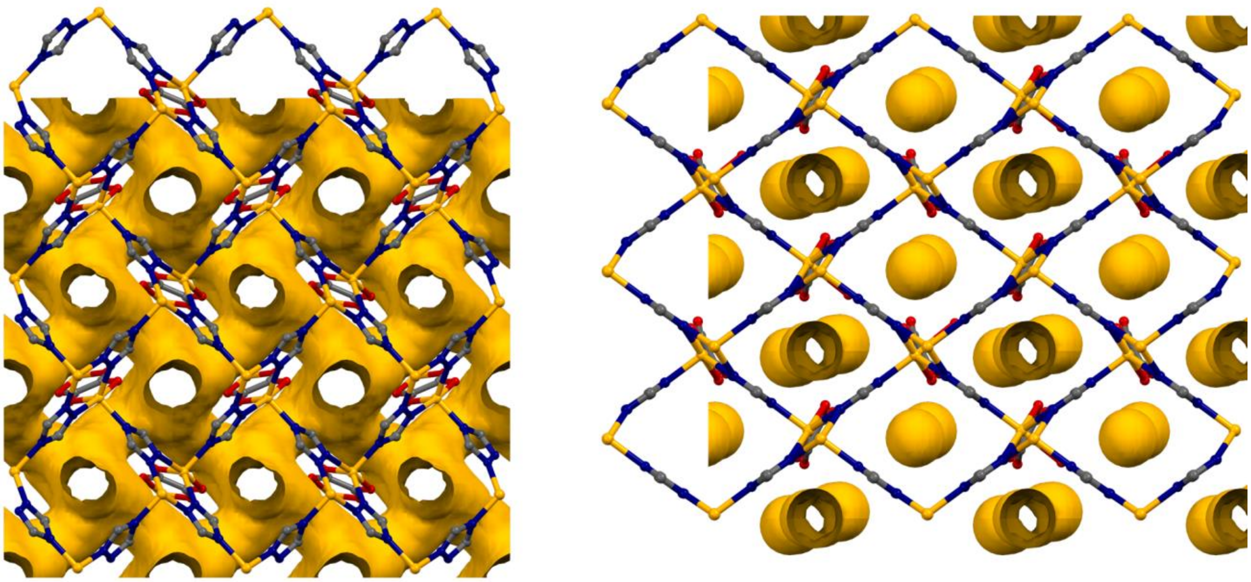

Figure S8 *Solvent-accessible voids in α-CALF-20 (left) and γ-CALF-20 (right) calculated with a probe radius of 1.3 Å viewed along the* x *axis. Hydrogen atoms have been omitted for clarity.*

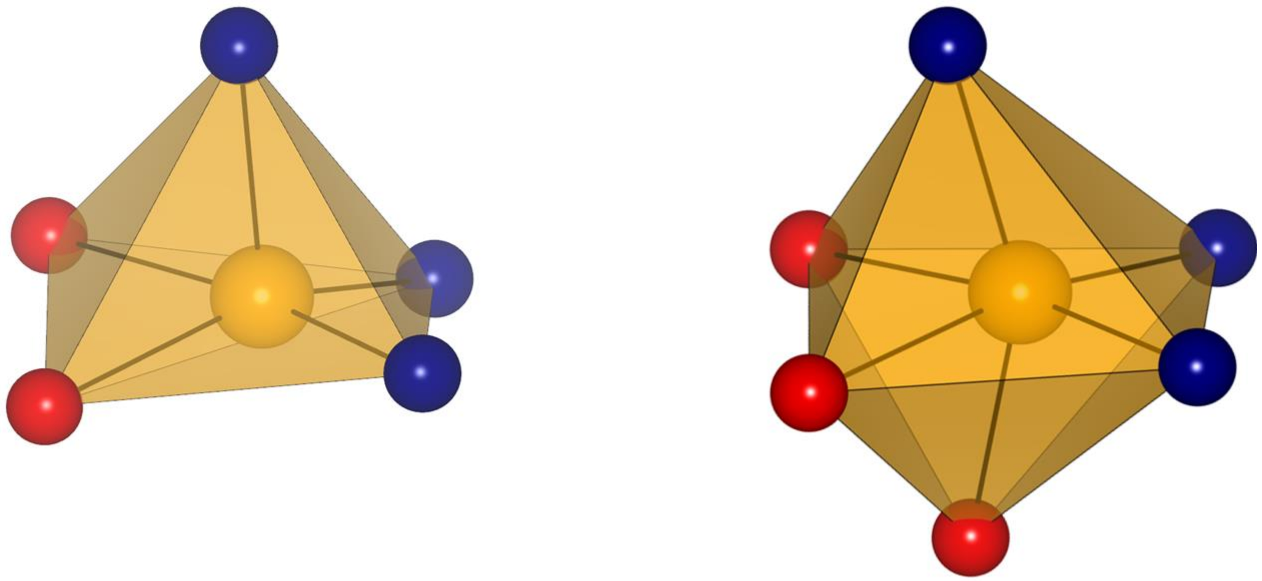

Figure S9 *Comparison of coordination spheres of* Zn^{2+} *centers simultaneously present in γ-CALF-20: the five-coordinated (left) and the six-coordinated (right) containing a water molecule.*

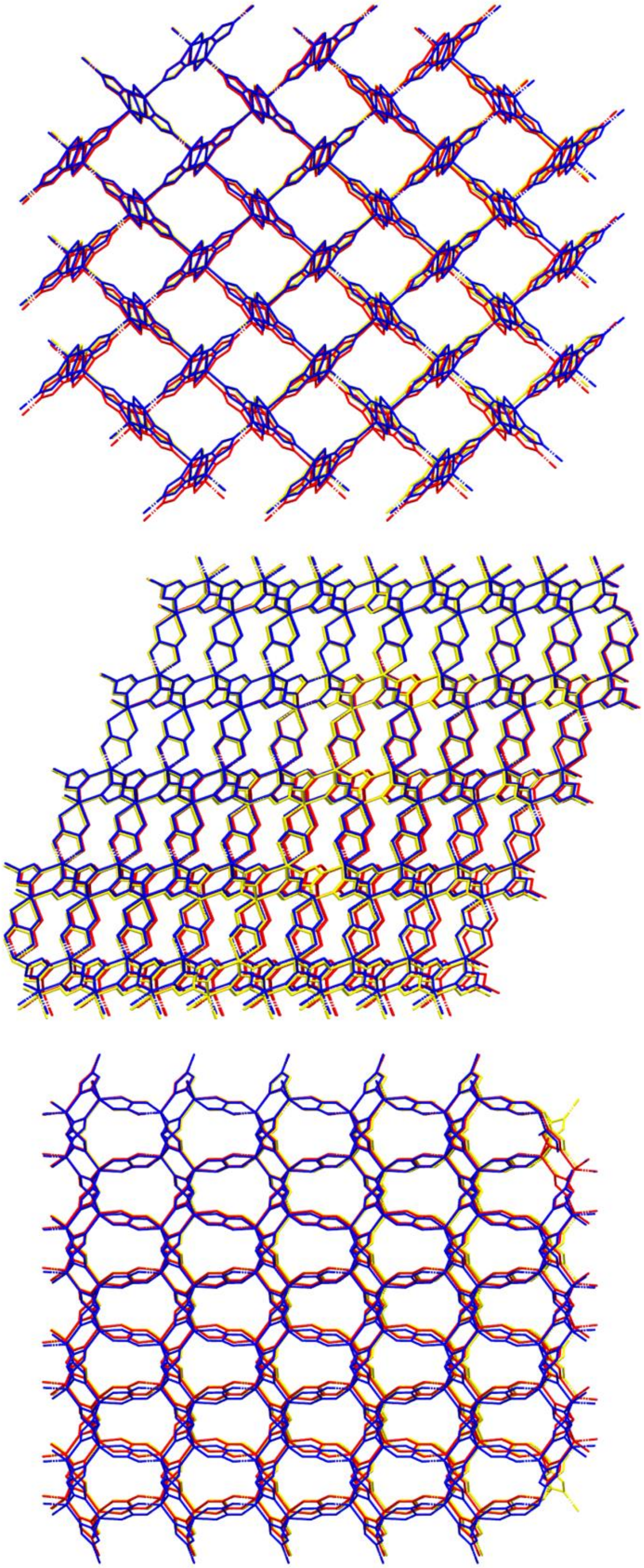

Figure S10 *Overlay of α-CALF-20 (red), α-CALF-20-act (yellow), and α-CALF-20-CO_2 (blue) structures in a view along the* x *(top),* y *(center), and* z *(bottom) axes.*

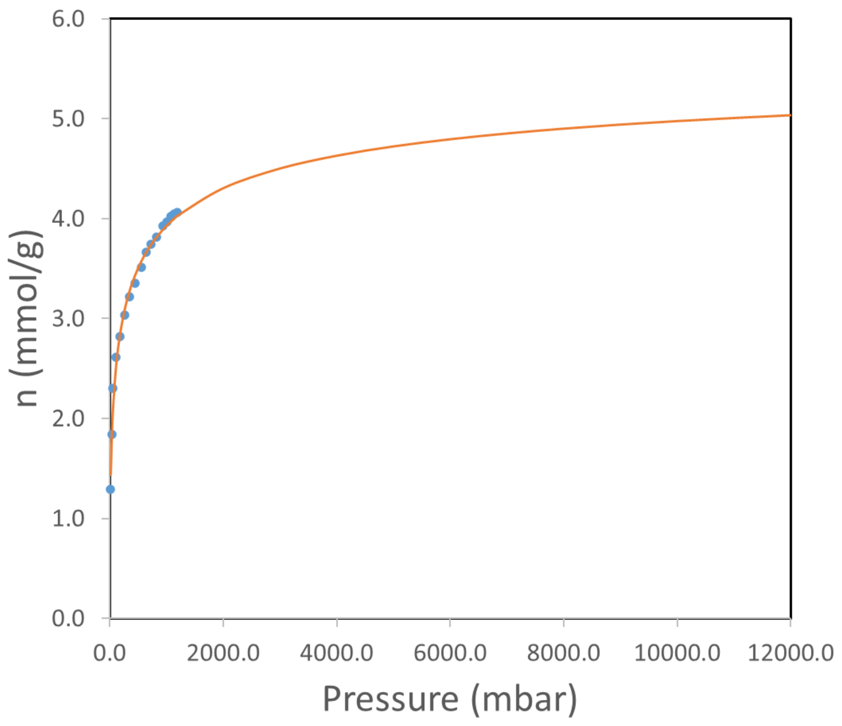

Figure S11 *The extrapolated* CO_2 *adsorption isotherm based on the data reported by Lin et al.*[2]

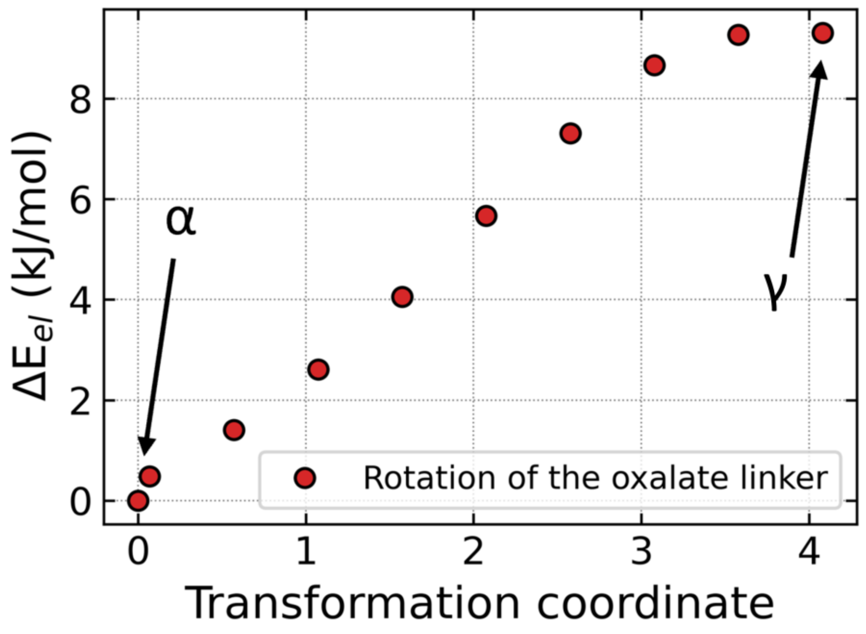

Figure S12 *SS-NEB analysis of the transformation between α-CALF-20 and γ-CALF-20 showing no transition state without the presence of water molecules in the pores.*

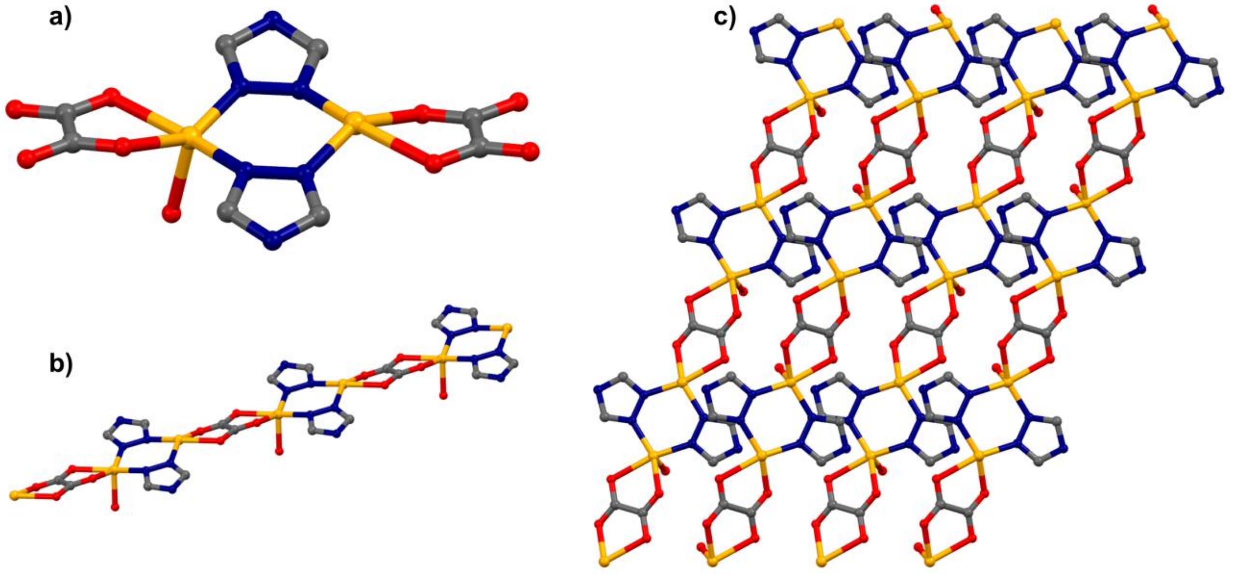

Figure S13 *a) The predicted crystal structure of δ-CALF-20, b) a chain formed by zinc cations coordinated to triazolate and oxalate, c) view of the crystal structure along the* y *axis. Hydrogen atoms have been omitted for clarity.*

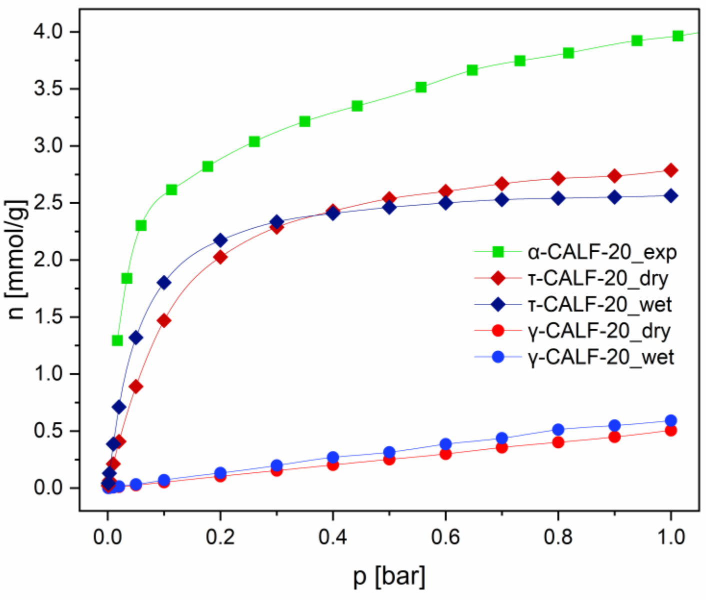

Figure S14 *CO_2 adsorption isotherms for α-CALF-20 (determined experimentally by Lin et al.[2]), τ-CALF-20, and γ-CALF-20 (determined theoretically for both phases without the presence of coordinated water molecules [$Zn_2(ox)(trz)_2$] [dry] and with water included in the model [$Zn_2(ox)(trz)_2(H_2O)$] [wet]).*

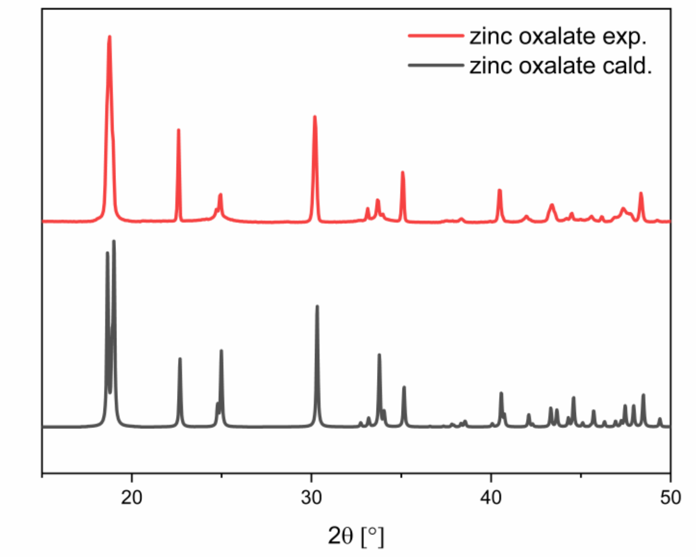

Figure S15 *P-XRD patterns of synthesized and calculated zinc oxalate dihydrate.*

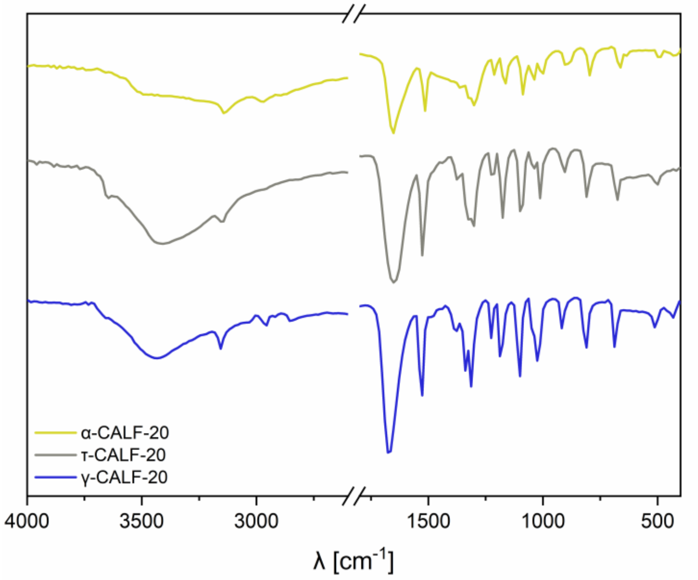

Figure S16 *FT-IR spectra of three CALF-20 phases.*

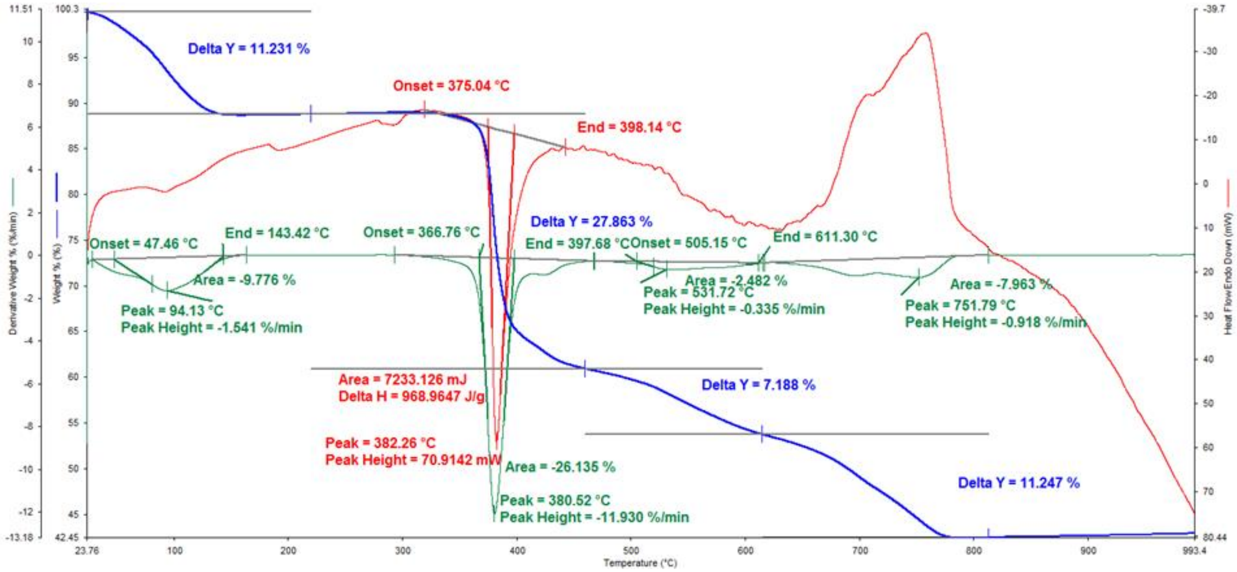

Figure S17 *The thermogravimetric analysis (blue) and differential scanning calorimetry (red) curves of bulk γ-CALF-20.*

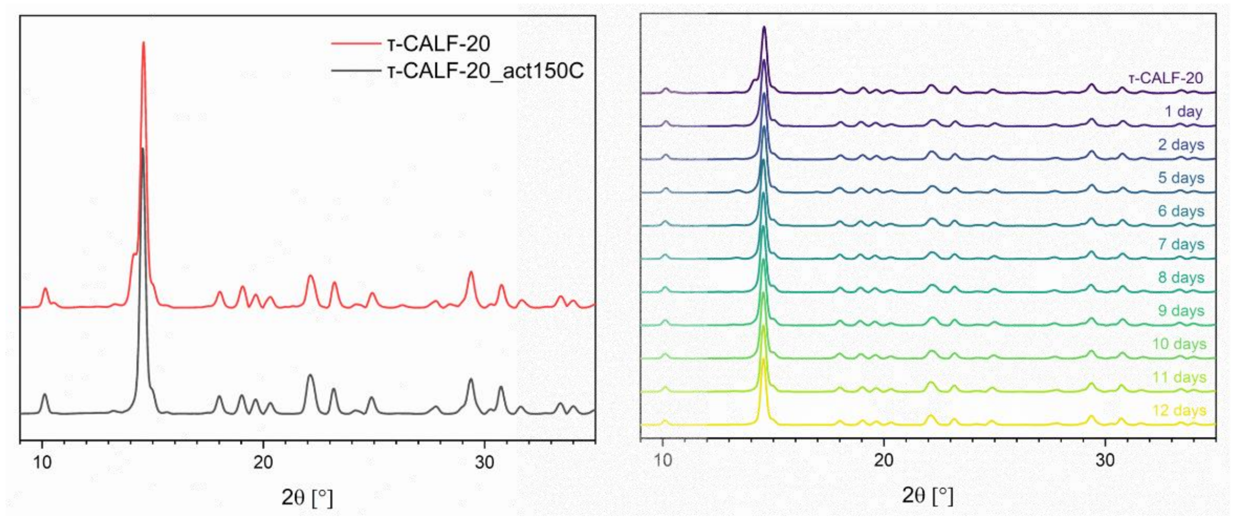

Figure S18 *Experimental powder patterns of τ-CALF-20 after heating at 150 °C under vacuum for four hours (left) and heating at 80 °C in air for twelve days (right).*

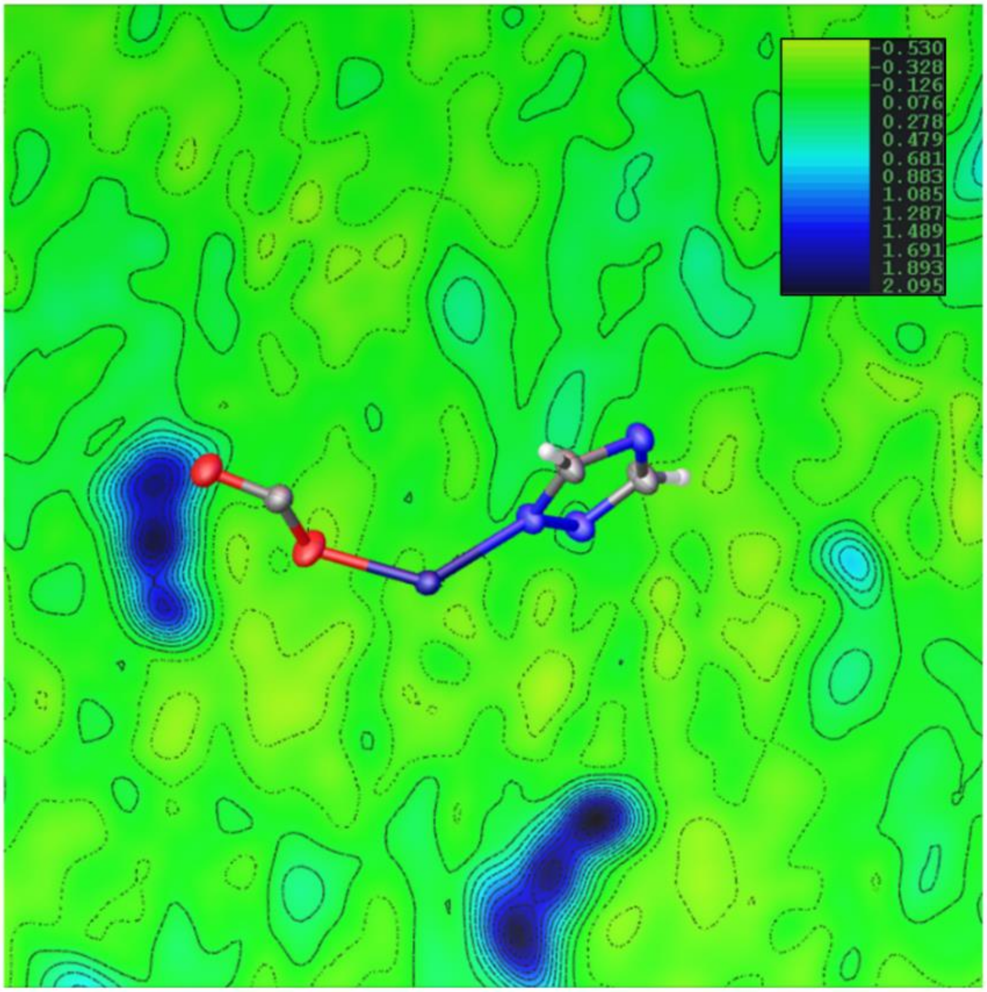

Figure S19 *Difference (Fo-Fc) electron density map for α-CALF-20-CO_2.*

Tables

Table S1 Crystallographic data for α-CALF-20, γ-CALF-20, τ-CALF-20, α-CALF-20-act, and α-CALF-20-CO_2.

phase	**α-CALF-20**	**γ-CALF-20**	**τ-CALF-20**	**α-CALF-20-act**	**α-CALF-20-CO_2**
Empirical formula	$C_3H_2N_3O_2Zn$	$C_6H_4N_6O_5Zn_2$	$C_6H_4N_6O_5Zn_2$	$C_3H_2N_3O_2Zn$	$C_7H_4N_6O_6Zn_2$
Formula weight [g/mol]	177.45	370.89	370.89	177.45	398.90
Temperature [K]	296(2)	140.10(14)	296(2)	296(2)	296(2)
Crystal system	monoclinic	monoclinic	monoclinic	monoclinic	monoclinic
Space group	$P2_1/c$	$P2_1/c$	$P2_1/c$	$P2_1/c$	$P2_1/c$
a [Å]	8.9375(2)	9.3919(5)	9.3052(4)	8.9765(2)	8.9367(3)
b [Å]	9.7321(2)	7.5264(4)	7.9498(3)	9.7142(3)	9.8435(3)
c [Å]	9.5429(2)	10.2078(6)	10.0430(5)	9.4546(3)	9.4623(3)
α [°]	90	90	90	90	90
β [°]	115.679(1)	110.078(6)	110.117(5)	116.217(1)	116.636(1)
γ [°]	90	90	90	90	90
Volume [$Å^3$]	748.07(3)	677.71(7)	697.60(6)	739.62(4)	744.04(4)
Z	4	2	2	4	2
ρ_{calc} [g/cm^3]	1.576	1.818	1.766	1.594	1.781
μ [mm^{-1}]	4.111	4.627	4.495	4.158	4.321
F(000)	348.0	364.0	364.0	348.0	392.0
Crystal size [mm^3]	0.25 × 0.25 × 0.11	0.152 × 0.11 × 0.096	0.205 × 0.199 × 0.072	0.25 × 0.25 × 0.11	0.25 × 0.25 × 0.11
Radiation	CuKα (λ = 1.54178)	CuKα (λ = 1.54178)	CuKα (λ = 1.54178)	CuKα (λ = 1.54178)	CuKα (λ = 1.54178)
2Θ range for data collection [°]	10.984 to 144.682	10.028 to 152.552	10.124 to 152.64	13.86 to 144.666	13.804 to 144.804
Index ranges	$-10 \leq h \leq 11$, $-12 \leq k \leq 10$, $-11 \leq l \leq 11$	$-11 \leq h \leq 11$, $-9 \leq k \leq 9$, $-12 \leq l \leq 12$	$-11 \leq h \leq 9$, $-9 \leq k \leq 10$, $-12 \leq l \leq 12$	$-11 \leq h \leq 11$, $-11 \leq k \leq 10$, $-11 \leq l \leq 11$	$-11 \leq h \leq 11$, $-12 \leq k \leq 11$, $-11 \leq l \leq 11$
Reflections collected	8679	7033	6091	8555	9368
Independent reflections	1465 [R_{int} = 0.0248, R_{sigma} = 0.0192]	1410 [R_{int} = 0.0391, R_{sigma} = 0.0232]	1433 [R_{int} = 0.0393, R_{sigma} = 0.0350]	1437 [R_{int} = 0.0303, R_{sigma} = 0.0235]	1451 [R_{int} = 0.0282, R_{sigma} = 0.0201]
Data/restraints/parameters	1465/0/90	1410/0/91	1433/0/99	1437/0/90	1451/2/114
Goodness-of-fit on F^2	1.093	1.084	1.105	1.146	1.172
Final R indexes [I>=2σ (I)]	R_1 = 0.0212, wR_2 = 0.0586	R_1 = 0.0475, wR_2 = 0.1284	R_1 = 0.0442, wR_2 = 0.1068	R_1 = 0.0292, wR_2 = 0.0796	R_1 = 0.0298, wR_2 = 0.920
Final R indexes [all data]	R_1 = 0.0226, wR_2 = 0.0590	R_1 = 0.0519, wR_2 = 0.1329	R_1 = 0.0627, wR_2 = 0.1395	R_1 = 0.0313, wR_2 = 0.0809	R_1 = 0.0323, wR_2 = 0.0926
Largest diff. peak/hole [e Å^{-3}]	0.25/-0.42	1.09/-0.95	0.46/-0.93	0.51/-0.43	0.74/-0.37

Table S2 Selected bond distances [Å] and angles [°] for α-CALF-20.

bond distances [Å]			
Zn1-O1	2.032(1)	Zn1-O2[a]	2.199(2)
Zn1-N1	2.014(2)	Zn1-N2[b]	2.095(2)
Zn1-N4[c]	2.030(2)		
bond angles [°]			
O1-Zn1-O2[a]	77.9(1)	O1-Zn1-N1	131.1(1)
O1-Zn1-N2[b]	86.9(1)	O1-Zn1-N4[c]	114.3(1)
O2[a]-Zn1-N1	88.0(1)	O2[a]-Zn1-N2[b]	164.7(1)
O2[a]-Zn1-N4[c]	89.7(1)	N1-Zn1-N2[b]	112.1(1)
N1-Zn1-N4[c]	112.1(1)	N2[b]-Zn1-N4[c]	97.7(1)

symmetry codes: [a]3-x,-y,2-z; [b]2-x,-y, 2-z; [c]2-x,-½+y,1.5-z

Table S3 Selected bond distances [Å] and angles [°] for γ-CALF-20.

bond distances [Å]			
Zn1-O1	2.085(4)	Zn1-O2[a]	2.197(3)
Zn1-O3	2.320(6)	Zn1-N1[b]	2.078(4)
Zn1-N3	2.027(4)	Zn1-N4[c]	2.081(4)
bond angles [°]			
O1-Zn1-O2[a]	77.0(1)	O1-Zn1-O3	74.0(3)
O1-Zn1-N1[b]	98.3(2)	O1-Zn1-N3	152.1(2)
O1-Zn1-N4[c]	91.3(1)	O2[a]-Zn1-O3	86.7(2)
O2[a]-Zn1-N1[b]	88.5(1)	O2[a]-Zn1-N3	89.4(1)
O2[a]-Zn1-N4[c]	168.3(1)	O3-Zn1-N1[b]	171.7(2)
O3-Zn1-N3	81.0(3)	O3-Zn1-N4[c]	91.4(2)
N1[b]-Zn1-N3	105.6(2)	N1[b]-Zn1-N4[c]	92.0(2)
N3-Zn1-N4[c]	101.7(2)		

symmetry codes: [a]1-x,1-y,1-z; [b]-x,1.5-y,z-½; [c]-x,1-y,1-z

Table S4 Selected bond distances [Å] and angles [°] for τ-CALF-20.

bond distances [Å]			
Zn1-O1	2.062(5)	Zn1-O2[a]	2.178(4)
Zn1-O3	2.50(1)	Zn1-N1[b]	2.026(5)
Zn1-N2[c]	2.088(4)	Zn1-N4	2.051(5)
bond angles [°]			
O1-Zn1-O2[a]	77.9(2)	O1-Zn1-O3	68.6(3)
O1-Zn1-N1[b]	150.2(2)	O1-Zn1-N2[c]	89.5(2)
O1-Zn1-N4	100.9(2)	O2[a]-Zn1-O3	83.9(3)
O2[a]-Zn1-N1[b]	88.6(2)	O2[a]-Zn1-N2[c]	166.8(2)
O2[a]-Zn1-N4	91.4(2)	O3-Zn1-N1[b]	83.7(3)
O3-Zn1-N2[c]	88.2(3)	O3-Zn1-N4	169.2(3)
N1[b]-Zn1-N2[c]	101.0(2)	N1[b]-Zn1-N4	105.9(2)
N2[c]-Zn1-N4	94.5(2)		

symmetry codes: [a]1-x,1-y,1-z; [b] x,1.5-y, z-½; [c]2-x,y-½,1.5-z

Table S5 Comparison of unit cell parameters of α-CALF-20, β-CALF-20 reported by Chen *et al.*[3], τ-CALF-20, and γ-CALF-20.

	**α-CALF-20**	**β-CALF-20**	**τ-CALF-20**	**γ-CALF-20**
space group	$P2_1/c$	$P2_1/c$	$P2_1/c$	$P2_1/c$
a [Å]	8.9735(2)	9.2788(4)	9.3052(4)	9.3919(5)
b [Å]	9.7321(1)	7.9340(3)	7.9498(3)	7.5264(4)
c [Å]	9.5429(2)	10.0393(4)	10.0430(5)	10.2078(6)
α [°]	90	90	90	90
β [°]	115.6790(10)	109.893(5)	110.117(5)	110.078(6)
γ [°]	90	90	90	90
Volume [$Å^3$]	748.07(3)	695.0(5)	697.60(6)	677.7

Table S6 Unit cell parameters of α-CALF-20, γ-CALF-20, and δ-CALF-20 obtained from the DFT calculations.

	**α-CALF-20**	**γ-CALF-20**	**δ-CALF-20**
a [Å]	9.0773	9.70283	9.4340
b [Å]	9.85910	7.85002	6.4080
c [Å]	9.38800	10.01112	10.4900
α [°]	90.00	90.00	90.00
β [°]	117.49	114.57	113.55
γ [°]	90.00	90.00	90.00
Volume [$Å^3$]	745.3181	693.4631	581.3229

References

(1) Wei, Y. *et al.* Efficient Xe Selective Separation from Xe/Kr/N2 Mixtures over a Microporous CALF-20 Framework. *RSC Adv.* **2022**, *12*, 18224–18231. https://doi.org/10.1039/D2RA02768B.

(2) Lin, J.-B. *et al.* A Scalable Metal-Organic Framework as a Durable Physisorbent for Carbon Dioxide Capture. *Science* **2021**, *374*, 1464–1469. https://doi.org/10.1126/science.abi7281.

(3) Chen, Z. *et al.* Humidity-Responsive Polymorphism in CALF-20: A Resilient MOF Physisorbent for CO_2 Capture. *ACS Materials Lett.* **2023**, 2942–2947. https://doi.org/10.1021/acsmaterialslett.3c00930.
